# Ovarian-Adnexal Reporting Data System Magnetic Resonance Imaging (O-RADS MRI) Score for Risk Stratification of Sonographically Indeterminate Adnexal Masses

**DOI:** 10.1001/jamanetworkopen.2019.19896

**Published:** 2020-01-24

**Authors:** Isabelle Thomassin-Naggara, Edouard Poncelet, Aurelie Jalaguier-Coudray, Adalgisa Guerra, Laure S. Fournier, Sanja Stojanovic, Ingrid Millet, Nishat Bharwani, Valerie Juhan, Teresa M. Cunha, Gabriele Masselli, Corinne Balleyguier, Caroline Malhaire, Nicolas F. Perrot, Elizabeth A. Sadowski, Marc Bazot, Patrice Taourel, Raphaël Porcher, Emile Darai, Caroline Reinhold, Andrea G. Rockall

**Affiliations:** 1 Service de Radiologie, Hôpital Tenon, Assistance Publique–Hôpitaux de Paris, Sorbonne Université, Paris, France; 2 Institute for Computing and Data Sciences, Sorbonne Université, Paris, France; 3 American College of Radiology, Ovarian-Adnexal Reporting and Data System Magnetic Resonance Imaging Committee; 4 Service d’Imagerie de la Femme, Centre Hospitalier de Valenciennes, Valenciennes, France; 5 Institut Paoli Calmettes, Marseille, France; 6 Hospital da Luz, Lisboa, Portugal; 7 Department of Radiology, Hôpital Européen Georges Pompidou, Assistance Publique-Hôpitaux de Paris, Université Paris-Descartes, Paris, France; 8 Centre for Radiology, Clinical Centre of Vojvodina, Medical Faculty, University of Novi Sad, Novi Sad, Serbia and Montenegro; 9 Lapeyronie Hospital, University of Montpellier, Montpellier, France; 10 Department of Radiology, Imperial College Healthcare NHS Trust, London, United Kingdom; 11 Hôpital de la Timone, Marseille, France; 12 Department of Radiology, Instituto Português de Oncologia de Lisboa Francisco Gentil, Lisboa, Portugal; 13 Department of Radiology, Umberto I Hospital, Sapienza University Roma, Rome, Italy; 14 Institut Gustave Roussy, Paris, France; 15 Institut Curie, Paris, France; 16 Centre Pyramides, Paris, France; 17 University of Wisconsin, Madison, Wisconsin; 18 Centre of Research in Epidemiology and Statistics Sorbonne Paris Cité, Institute national de la santé et de la recherche médicale, Joint Research Unit 1153, Paris, France; 19 Service de Gynecologie et Obstetrique et Médecine de la Reproduction, Hôpital Tenon, Assistance Publique–Hôpitaux de Paris, Hôpitaux Univesitaires Est Parisien, Paris, France; 20 Faculté de Médecine Pierre et Marie Curie, Sorbonne Université, Paris, France; 21 Department of Medical Imaging, McGill University Health Centre, Montreal, Quebec, Canada; 22 Division of Cancer and Surgery, Faculty of Medicine, Imperial College London, United Kingdom

## Abstract

**Importance:**

Approximately one-quarter of adnexal masses detected at ultrasonography are indeterminate for benignity or malignancy, posing a substantial clinical dilemma.

**Objective:**

To validate the accuracy of a 5-point Ovarian-Adnexal Reporting Data System Magnetic Resonance Imaging (O-RADS MRI) score for risk stratification of adnexal masses.

**Design, Setting, and Participants:**

This multicenter cohort study was conducted between March 1, 2013, and March 31, 2016. Among patients undergoing expectant management, 2-year follow-up data were completed by March 31, 2018. A routine pelvic MRI was performed among consecutive patients referred to characterize a sonographically indeterminate adnexal mass according to routine diagnostic practice at 15 referral centers. The MRI score was prospectively applied by 2 onsite readers and by 1 reader masked to clinical and ultrasonographic data. Data analysis was conducted between April and November 2018.

**Main Outcomes and Measures:**

The primary end point was the joint analysis of true-negative and false-negative rates according to the MRI score compared with the reference standard (ie, histology or 2-year follow-up).

**Results:**

A total of 1340 women (mean [range] age, 49 [18-96] years) were enrolled. Of 1194 evaluable women, 1130 (94.6%) had a pelvic mass on MRI with a reference standard (surgery, 768 [67.9%]; 2-year follow-up, 362 [32.1%]). A total of 203 patients (18.0%) had at least 1 malignant adnexal or nonadnexal pelvic mass. No invasive cancer was assigned a score of 2. Positive likelihood ratios were 0.01 for score 2, 0.27 for score 3, 4.42 for score 4, and 38.81 for score 5. Area under the receiver operating characteristic curve was 0.961 (95% CI, 0.948-0.971) among experienced readers, with a sensitivity of 0.93 (95% CI, 0.89-0.96; 189 of 203 patients) and a specificity of 0.91 (95% CI, 0.89-0.93; 848 of 927 patients). There was good interrater agreement among both experienced and junior readers (κ = 0.784; 95% CI, 0.743-0824). Of 580 of 1130 women (51.3%) with a mass on MRI and no specific gynecological symptoms, 362 (62.4%) underwent surgery. Of them, 244 (67.4%) had benign lesions and a score of 3 or less. The MRI score correctly reclassified the mass origin as nonadnexal with a sensitivity of 0.99 (95% CI, 0.98-0.99; 1360 of 1372 patients) and a specificity of 0.78 (95% CI, 0.71-0.85; 102 of 130 patients).

**Conclusions and Relevance:**

In this study, the O-RADS MRI score was accurate when stratifying the risk of malignancy in adnexal masses.

## Key Points

**Question:**

Is the Ovarian-Adnexal Reporting Data System Magnetic Resonance Imaging (O-RADS MRI) score accurate for stratifying the risk of malignancy of sonographically indeterminate adnexal masses?

**Findings:**

In this multicenter cohort study that included 1340 women, the O-RADS MRI score had a sensitivity of 0.93 and a specificity of 0.91.

**Meaning:**

Applying this score in clinical practice may allow a tailored, patient-centered approach for adnexal masses that are sonographically indeterminate, preventing unnecessary surgery, less extensive surgery, or fertility preservation when appropriate, while ensuring preoperative detection of lesions with a high likelihood of malignancy.

## Teaser

This cohort study validates the accuracy of the 5-point Ovarian-Adnexal Reporting Data System Magnetic Resonance Imaging (O-RADS MRI) score for risk stratification of adnexal masses.

## Introduction

Adnexal masses are common, resulting in a significant clinical workload related to diagnostic imaging, surgery, and pathology.^1,2^ Most adnexal masses are benign, and most masses can be accurately categorized as benign or malignant on ultrasonography.^3,4^ However, between 18% and 31% of adnexal masses remain indeterminate following ultrasonography using International Ovarian Tumor Analysis (IOTA) Simple Rules or other ultrasonography scoring systems.^3,4^ Moreover, this prevalence may be an underestimation, given that many studies only report the cases with available surgical reference standards.^4^ There are very limited data on patients who undergo only imaging and clinical follow-up. In a prospective external validation of the IOTA Simple Rules^5^ among 666 women, 362 women (54.4%) underwent surgery, 309 of whom (85.4%) had benign masses. The authors reported that, among 304 patients (45.6%) who underwent expectant management, 71 patients (23.4%) experienced disappearance of the mass, and 233 (76.6%) had a persistent mass on imaging follow-up that was considered benign after 1 year of follow-up.

Percutaneous biopsy of a suspicious adnexal mass is not advised because of the risk of potentially upstaging a confined early-stage ovarian cancer or because of the risk of sampling error, resulting in a missed cancer diagnosis. As a result, despite the low rate of malignant adnexal masses discovered at ultrasonography (ie, 8%-20%),^5,6^ a significant number of women with sonographically indeterminate but benign adnexal masses undergo potentially unnecessary or inappropriately extensive surgical interventions.^7,8^ This increases the risk of loss of fertility as well as morbidity, as reported in the 2 largest ovarian cancer screening trials.^7,8^ Conversely, some women with an indeterminate adnexal mass undergo initial, limited, noncancer surgery and are found to have ovarian cancer, with a risk of suboptimal initial cytoreductive surgery and significantly poorer outcomes.^9^

Thus, preoperative characterization and risk stratification of indeterminate adnexal masses are unmet clinical needs. A validated scoring system that standardizes imaging reports and categorizes the risk of malignant neoplasm in these women would be useful as a triage test to decide whether surgery is appropriate and, if so, the extent of surgery required. This could potentially reduce unnecessary or overextensive surgery. In the literature, various scoring systems have been developed based on clinical, biochemical (eg, cancer antigen 125 [CA 125] or human epididymis protein 4 [HE 4] levels), and ultrasonographic criteria.^10,11^ Nevertheless, a significant subgroup of adnexal masses remain indeterminate despite optimal sonographic risk assessment, hampering treatment planning.^12,13^ A magnetic resonance imaging (MRI) scoring system was developed in a retrospective single-center study^14^ among a cohort of 497 patients with indeterminate adnexal masses at ultrasonography. This MRI-based score consisted of 5 categories according to the positive likelihood ratio for a malignant neoplasm.^14^ The score was based on MRI features with high positive and high negative predictive values in distinguishing benign from malignant masses that were considered indeterminate on ultrasonography. However, the score warrants validation from a multicenter study among a large cohort of women.

Therefore, the primary objective of this study was to test the score for risk stratification in women referred for an MRI of sonographically indeterminate adnexal masses in a large, prospective, multicenter clinical study. The findings provide the evidence to support the publication of the Ovarian-Adnexal Reporting Data System Magnetic (O-RADS) MRI score version 1.

## Methods

This prospective multicenter cohort study was conducted between March 1, 2013, and March 31, 2018. Participant enrollment took place between March 1, 2013, and March 31, 2016, with 2-year follow-up among 362 of 1340 patients (27.0%) undergoing expectant management, which was completed by March 31, 2018. Recruitment was undertaken in 15 centers, each with a principal investigator from the European Society of Urogenital Radiology Female Pelvic Imaging working group (eAppendix 1 in the Supplement). According to French regulations at the time of study initiation, the study was approved by the Comité Consultatif sur le Traitement de l'Information en matière de Recherche dans le domaine de la Santé. In addition, the protocol was approved by the ethics committee of each participating site. All participating women provided written informed consent. The study protocol appears in eAppendix 2 of the Supplement. This study followed the Strengthening the Reporting of Observational Studies in Epidemiology (STROBE) reporting guideline.

### Study Population

Consecutive women older than 18 years who were referred to a study center for MRI to characterize a sonographically indeterminate adnexal mass were invited to participate. Exclusion criteria were pregnancy or any contraindication to MRI (eAppendix 3 in the Supplement).

### MRI Acquisition and Analysis

Each patient underwent a routine pelvic MRI (1.5 T or 3 T), including morphological sequences (ie, T2-weighted; T1-weighted, with and without fat suppression; and T1-weighted after gadolinium injection) and functional sequences (ie, perfusion-weighted and diffusion-weighted sequences). If no adnexal mass was present on T2-wieghted and T1-weighted sequences, functional sequences and gadolinium injection were not mandatory (eAppendix 4 in the Supplement).

The patients’ medical records were reviewed, and gynecological symptoms and ultrasonographic findings were recorded. The quality of the ultrasonography report was recorded using widely used standardized criteria (eAppendix 3 in the Supplement). Levels of CA 125 were recorded, if available. An experienced radiologist (ie, with >10 years of gynecological MRI expertise) (I.T.-N, E.P., A.J.-C., A.G., L.S.F., S.S., I.M., N.B, V.J., T.M.C., G.M, C.B., C.M., N.F.P., M.B., P.T., and A.G.R.) and a junior radiologist (ie, with 6-12 months of gynecological MRI expertise) read MRI scans prospectively and independently. They were unmasked to clinical and sonographic findings. Another experienced reader (with >10 years of gynecological MRI expertise) (I.T.-N., E.P., A.J.-C., V.J., and A.G.R.), masked to clinical and sonographic findings, read the MRI retrospectively. The readers characterized each mass according to a standardized lexicon and assigned a score.^14^ If there was no adnexal mass or if the origin of a pelvic mass was nonadnexal, readers were asked to assign a score of 1 to the adnexa and rate the nonadnexal mass as either suspicious or nonsuspicious for malignancy. The presence of solid tissue and its morphology (eg, enhancing solid papillary projection, thickened irregular septa, or the solid part of a mixed cystic solid or purely solid lesion) were evaluated. The reader then analyzed T2-weighted signal intensity within the solid tissue (ie, low or intermediate compared with the outer myometrium) and diffusion-weighted signal intensity within the solid tissue (ie, high diffusion-weighted signal intensity compared with serous fluid, eg, urine within bladder or cerebrospinal fluid). The reader classified the enhancement of the solid tissue using time intensity curve (TIC) classification.^14^ When TIC classification was not feasible, it was rated as TIC type 2. An MRI score of 2 was assigned if the reader diagnosed a purely cystic mass (ie, adnexal unilocular cyst with simple fluid and no solid tissue), a purely endometriotic mass (ie, adnexal unilocular cyst with endometriotic fluid and no internal enhancement), a purely fatty mass (ie, adnexal cyst with unilocular or multilocular fatty content and no solid tissue), if there was no wall enhancement, or if solid tissue was detected with homogeneous hypointense T2-weighted as well as homogeneous hypointense high b value of diffusion-weighted solid tissue (ie, a dark-dark pattern). An MRI score of 3 was assigned if the reader diagnosed an adnexal unilocular cyst with proteinaceous or hemorrhagic fluid that did not comply with endometriotic fluid signal intensity and no solid tissue, an adnexal multilocular cyst and no solid tissue, or an adnexal lesion with solid tissue that enhanced with a TIC type 1 on dynamic-contrast enhanced MRI (excluding dark-dark solid tissue). An MRI score of 4 was assigned if the reader diagnosed an adnexal lesion with solid tissue that enhanced with a TIC type 2 on dynamic-contrast enhanced MRI (excluding dark-dark solid tissue). An MRI score of 5 was assigned if the reader diagnosed an adnexal lesion with solid tissue that enhanced with a TIC type 3 on dynamic-contrast enhanced MRI or if peritoneal or omental thickening or nodules were detected. Presence of ascites was noted. Up to 3 pelvic masses per patient were analyzed. All MRI readers were masked to the final outcome.

### Reader Training

During study setup, a session of 30 anonymized MRI scans (acquired before the beginning of the study) were downloaded for a training session for all teams participating in the multicenter validation to learn how to apply the score. A standardized lexicon was used for interpretation.^14^

### Reference Standard

Patient management was decided by a multidisciplinary team according to standard clinical practice in each center. The final diagnosis recorded for each patient was based on histology or clinical follow-up; if the lesion did not disappear or decrease at imaging follow-up, a minimum of 24 months of observation was performed (with or without imaging) from the date of the study MRI. Borderline lesions were considered malignant. In cases that underwent clinical follow-up, the origin of the pelvic mass was confirmed if there was agreement by the 2 experienced readers. In cases of disagreement, a final decision was made by a consensus panel of 5 radiologists (with >10 years of gynecological MRI expertise) (I.T.-N., I.M., and P.T.) from 2 sites.

### Statistical Analysis

The study end point was the joint analysis of true-negative and false-negative rates according to the MRI score compared with the reference standard. The sample size was determined based on previous results^14^ to ensure that this study would have power of at least 90% to show a difference in diagnostic odds ratio between a score of 2 and 3 and between a score of 4 and 5. A total sample size of 1340 patients would ensure a probability of at least 95% to obtain the required 569, 250, 52, and 51 patients with MRI scores of 2, 3, 4, and 5, respectively,^15^ assuming that 6% of patients would have lesions classified as a score of 1 and 10% of patients would be lost to follow-up.

For statistical analysis, the MRI score was matched to the reference standard. These analyses used the prospective, experienced reader’s rating.

Diagnostic accuracy was evaluated both at the patient level and at the lesion level in terms of positive likelihood ratios (PLRs) and negative likelihood ratios (NLRs) for malignant masses. In addition, sensitivities, specificities, positive predictive values (PPVs), and negative predictive values (NPVs) were computed for dichotomized scores (ie, score of 2 and 3 [benign] vs score of 4 and 5 [malignant], according to predefined cutoff at 3 for the score).

To evaluate interobserver agreement, we used receiver operating characteristic curve analysis and compared the area under receiver operating characteristic curves between experienced and junior readers.^16^ We also computed weighted quadratic κ coefficients.^17^

Patients lost to follow-up (130 [9.7%]), patients for whom MRI failed to be completed (9 [0.7%]), and patients who withdrew consent (7 [0.5%]) were excluded from analyses. Among patients who were lost to follow-up, subjective assessment by experienced readers was indeterminate, borderline, or invasive in 12 of 130 patients (9.2%).

Estimates are provided with their 95% CIs. A 2-tailed *P* < .05 was considered statistically significant. Statistical analyses were performed using MedCalc version 9.3.0.0 (MedCalc Software) and R version 3.5.0 (R Project for Statistical Computing).

## Results

### Patients and Lesions

**Figure 1.**
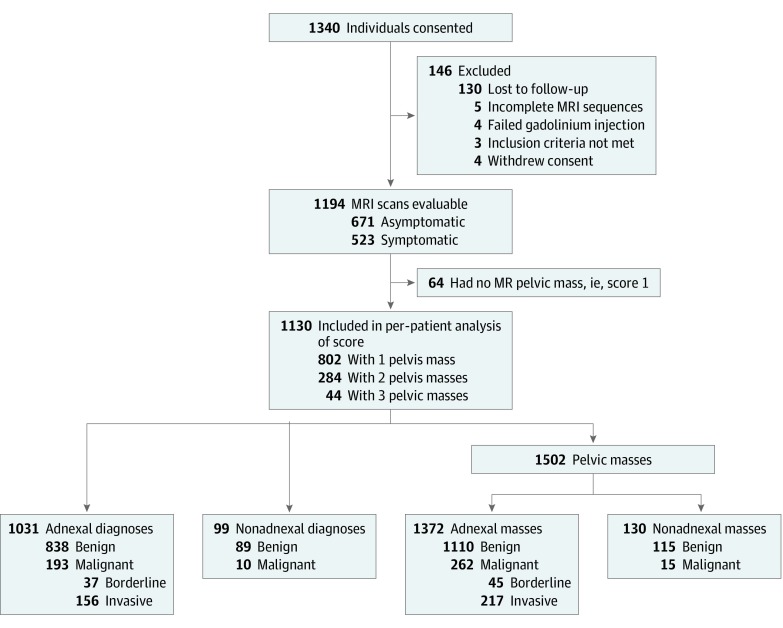
Study Population Flow Diagram MR indicates magnetic resonance; MRI, magnetic resonance imaging.

Overall, 1340 patients were enrolled in the study. The mean (range) age was 49 (18-96) years. The final, evaluable population included 1194 patients (89.1%), after 130 (9.7%) patient withdrawals (Figure 1). Of the included patients, 64 (5.4%) were found not to have a pelvic mass. The remaining 1130 patients (94.6%) had a total of 1502 pelvic masses.

Patient characteristics and clinical symptoms are described in Table 1. Patients were referred for indeterminate adnexal masses based on the results of a pelvic ultrasonograph with an issued report rated as high quality, with scores equal to or greater than 5 to 7 in 950 of 1194 patients (79.6%) (eAppendix 3 in the Supplement). Solid tissue was suspected at ultrasonography in 523 women (43.8%), including 166 malignant lesions (11.1%) and 357 benign lesions (23.8%). Ultrasonographic size of the mass was greater than 6 cm in 337 women (28.2%), including 95 malignant lesions (6.3%) and 242 benign lesions (16.1%).

**Table 1.** Population Characteristics

Characteristics	No. (%)
**Personal History (n = 1194)**	
Menopausal	511 (42.8)
History of pelvic surgery	364 (30.5)
History of adnexal surgery	134 (11.2)
History of infertility	94 (7.9)
History of breast or ovarian cancer	126 (10.5)
Known *BRCA*1/2 carriers	13 (1.1)
**Clinical Presentation (n = 1194)**	
Pelvic pain	384 (32.2)
Vaginal bleeding	60 (5.0)
Palpable mass or increasing abdominal volume	10 (0.8)
Urinary symptoms	8 (0.7)
Amenorrhea	6 (0.5)
Constipation or diarrhea	2 (0.2)
Combination of previously mentioned symptoms	53 (4.4)
None of these symptoms	671 (56.2)
**MRI Findings (n = 1194)**	
0 Lesions	64 (5.4)
1 Lesion	802 (67.2)
2 Lesions	284 (23.8)
3 Lesions	44 (3.7)
**Management (n = 1130)**	
Primary surgery	719 (63.6)
Secondary surgery	
After initial follow-up	44 (3.9)
After primary chemotherapy	5 (0.4)
24 mo of clinical and/or imaging follow-up	362 (32)
Imaging follow-up	263 (23.3)
Disappearance	96 (8.4)
Decrease of the mass	15 (1.3)
Stability	152 (13.4)
Clinical follow-up, ie, stability^a^	99 (8.8)
**Origin of Pelvic Mass From Reference Standard**	
Masses, No.	1502
Adnexal	
Masses, No.	1372
Malignant, No./total, No. (%)	
Ovary	253/1223 (20.7)
Tubo-ovarian	9/125 (7.2)
Mesosalpinx	0/24
Nonadnexal	
Masses, No.	130
Malignant, No./total, No. (%)	
Uterus	4/72 (5.5)
Peritoneal^b^	3/41 (7.3)
Colorectal	5/5 (100)
Lymph node	2/2 (100)
Other, eg, schwannoma, arterial aneurysm	0/9
Urothelial	1/1 (100)

Abbreviation: MRI, magnetic resonance imaging.

^a^ The 99 women without imaging follow-up had 122 lesions with the following clinical diagnoses: 23 nonadnexal masses including 14 leiomyomas, 5 peritoneal cysts, 3 hematoma, and 1 Nabothian cyst; 26 serous cystadenomas; 24 functional cysts; 23 endometrioma; 9 cystadenofibroma; 5 ovarian fibroma; 5 hydrosalpinx; 4 mature cystic teratoma; 2 mucinous cystadenoma; and 1 paraovarian cyst.

^b^ This category excludes tubo-ovarian peritoneal carcinoma and includes other purely peritoneal diseases, such as pseudoperitoneal cysts.

Levels of CA 125 were available for 537 patients (44.9%), 398 (74.1%) of whom had benign and 139 (25.9%) of whom had malignant tumors. An elevated CA 125 level (ie, ≥35 U/mL; to convert to kU/L, multiply by 1.0) indicated malignant neoplasm as follows: sensitivity, 0.68 (95% CI, 0.61-0.76; 95 of 139 patients); specificity, 0.82 (95% CI, 0.78-0.86; 327 of 398 patients); PLR, 3.83 (95% CI, 3.02-4.87); NLR, 0.39 (95% CI, 0.30-0.49); PPV, 0.57 (95 of 166 patients); NPV, 0.88 (327 of 371 patients); and accuracy, 0.78 (422 of 537 patients).

A total of 915 women (76.7%) had MRI performed with a 1.5-T MRI scanner and 279 women (23.4%) with a 3-T MRI scanner using 3 different vendors (Siemens, Philips, GE Healthcare). Quality of MRI scans was considered good in 1160 of 1194 women (97.1%), with motion artifacts in 184 of 1194 (15.4%), but all scans remained diagnostic.

Of 1130 patients with a pelvic mass, 768 (67.9%) underwent surgery, and 362 (32.1%) underwent standard clinical follow-up (Table 1). Of those undergoing clinical follow-up, imaging was included for 263 women (72.6%).

There were 130 nonadnexal masses (8.6%) and 1372 adnexal masses (91.3%). The origins of nonadnexal masses are reported in Table 1. The prevalence of malignant neoplasms in the population of women with pelvic mass on MRI was 18.0% (203 of 1130).

### Analysis of the MRI Score at the Patient Level

#### Validation per Patient

##### Discrimination

The score yielded an area under the receiver operating characteristic curve of 0.961 (95% CI, 0.948-0.971) among experienced readers and 0.942 (95% CI, 0.927-0.955) among junior readers, with a higher performance for the experienced readers (*P* = .03) (eFigure in the Supplement). Among experienced and junior readers, a score of 4 or 5 suggested a malignant mass with a sensitivity of 0.93 (95% CI, 0.89-0.96; 189 of 203 patients) and 0.92 (95% CI, 0.87-0.95; 186 of 203 patients), respectively, and a specificity 0.91 (95% CI, 0.89-0.93; 848 of 927 patients) and 0.90 (95% CI, 0.88-0.91; 833 of 927 patients), respectively. The prevalence of masses that remained indeterminate on MRI (ie, score of 4) remained low, with 122 (10.8%) among experienced readers and 141 (12.5%) among junior readers.

##### Performance per Patient

Among 91 women assigned a score of 1, 78 women (85.7%) with nonadnexal masses were subjectively rated nonsuspicious, and 13 women (14.3%) were subjectively rated suspicious by the readers (Table 2 and Table 3).^18^ All nonsuspicious masses were benign, and suspicious masses were highly indicative of a malignant tumor (PLR, 15.22; 95% CI, 4.23-54.82) (Table 3). Of the 13 women with at least 1 suspicious mass, 10 women (76.9%) had malignant tumors, and 3 (23.1%) had benign leiomyomas with degeneration.

**Table 2.** Diagnostic Performance of the Magnetic Resonance Imaging Score^a^

Characteristic	Score (95% CI)	
	Experienced Readers	Junior Readers
Performance, No.		
True-positive result	189	186
False-negative result	14	17
True-negative result	848	833
False-positive result	79	94
Sensitivity	0.93 (0.89-0.96)	0.92 (0.87-0.95)
Specificity	0.91 (0.89-0.93)	0.90 (0.88-0.91)
Likelihood ratio		
Positive	10.90 (8.82-13.50)	9.04 (7.43-11.00)
Negative	0.08 (0.05-0.13)	0.09 (0.06-0.15)
Predictive value		
Positive	0.71 (0.65-0.76)	0.66 (0.61-0.72)
Negative	0.98 (0.97-0.99)	0.98 (0.97-0.99)
Accuracy	0.92 (0.90-0.93)	0.90 (0.88-0.92)
Diagnostic odds ratio	145.00 (80.30-261.00)	97.00 (56.50-166.00)

^a^ A total of 1130 magnetic resonacing imaging scans were scored. Sensitivities, specificities, and positive and negative predictive values were computed for dichotomized scores (ie, score of 2 and 3 [benign] vs score of 4 and 5 [malignant] or score 1 [nonadnexal mass rated suspicious]). A total of 203 of 1130 patients (18.0%) had at least 1 malignant mass of adnexal or nonadnexal origin.

**Table 3.** Experienced Readers’ MRI Scores Prospectively Assigned to 1130 Patients With Pelvic Masses

MRI Score	Patients, No.	Positive Likelihood of Malignant Tumor (95% CI)^a^	Patients, No./Total No. (%)^b^		
			With Borderline Tumors	With Invasive Tumors	With Malignant Tumors^c^
1	91	0.53 (0.30-1.07)	0	10/91 (10.9)	10/91 (10.9)
Nonsuspicious nonadnexal	78	0 (0-0.16)	0	0	0
Suspicious nonadnexal	13	15.22 (4.23.54.82)	0	10/13 (76.9)	10/13 (76.9)
2	571	0.01 (0-0.04)	2/571 (0.3)	0	2 (0.3)
3	213	0.27 (0.16-0.48)	8/213 (3.7)	4/213 (1.9)	12/213 (5.6)
No solid tissue	120	0.17 (0.06-0.45)	2/120 (1.7)	2/120 (1.7)	4/120 (3.3)
Solid tissue	93	0.46 (0.23-0.93)	6/93 (6.4)	2/93 (2.1)	8/93 (8.6)
4	122	4.42 (3.31-6.09)	20/122 (16.4)	40/122 (32.8)	60/122 (49.2)
Fatty content	14		0	0	0
No fatty content	108		20/108 (18.5)	40/108 (37.0)	60/108 (55.5)
5	133	38.81 (22.79-66.11)	7/133 (5.2)	112/133 (84.2)	119/133 (89.5)
Fatty content	6		0	1/6 (16.7)	1/6 (16.7)
No fatty content	127		7/127 (5.5)	111/127 (87.4)	118/127 (92.9)

Abbreviation: MRI, magnetic resonance imaging.

^a^ Confidence intervals obtained by bootstrapping.^18^

^b^ A total of 64 evaluable patients did not have a pelvic mass at the time of the MRI scan, and 91 patients had a nonadnexal mass.

^c^ Malignant includes borderline and invasive tumors.

Among 571 women assigned a score of 2, 569 (99.6%) had benign lesions (Table 3). Two premenopausal women (0.3%) had serous borderline tumors (false-negative rate, 0.3%) (Table 3).

Among 213 women assigned a score of 3, malignant tumors were found in 6 premenopausal women (2.8%) and 6 menopausal women (2.8%) (false-negative rate, 5.6%), including 4 women (33.3%) with masses containing no solid tissue (1 premenopausal woman [25.0%] and 3 menopausal women [75.0%]) (Table 3). Eight of 12 malignant tumors (75.0%) were borderline. All 12 fat-containing lesions were benign.

Among 122 women assigned a score of 4, 62 women (50.8%) had benign tumors, and 60 (49.2%) had malignant tumors, with a higher prevalence of invasive than borderline tumors (40 [32.8%] vs 20 [16.4%], respectively) (Table 3). All 14 fat-containing lesions were benign.

Among 133 women assigned a score of 5, 9 premenopausal (6.8%) and 5 menopausal women (3.8%) had benign lesions (false-positive rate, 10.5%), including 5 (35.7%) with mature teratomas, 2 (14.3%) with pelvic inflammatory disease, 2 (14.3%) with cystadenofibroma, 1 (7.1%) with Brenner tumors, 1 (7.1%) with serous cystadenoma, 1 (7.1%) with ovarian fibroma, 1 (7.1%) with struma ovarii, and 1 (7.1%) with a luteal cyst. Of the 6 fat-containing lesions, 5 (83.3%) were benign and 1 (16.7%) was a malignant germ cell (endodermal sinus) tumor (Table 3). The PLR for score 2 was 0.01; for score 3, 0.27; for score 4, 4.42; and for score 5, 38.81.

#### Potential Consequences for Management

In the study population, 580 of 1130 women (51.3%) with a mass on MRI and no specific gynecological symptoms underwent surgery (362 [62.4%]) or follow-up (218 [37.6%]). Based on the standard MRI report and management, 244 women (67.4%) (121 premenopausal and 123 menopausal) with benign lesions and a score of 3 or less or a nonadnexal mass rated as nonsuspicious underwent surgery, and 1 woman (0.5%) with an invasive tumor with a score of 4 or 5 underwent initial follow-up. Moreover, 8 women (2.2%) who underwent surgery had a score of 2 (2 [25.0%] with borderline tumors) or 3 (4 [50.0%] with borderline tumors and 2 [25.0%] with invasive tumors).

#### Reproducibility

The interrater agreement of the score between experienced and junior readers was substantial (κ = 0.784; 95% CI 0.743-0.824). Interrater agreement between experienced readers was also substantial (κ = 0.804; 95% CI, 0.764-0.844).

### Analysis of the Criteria Used in the MRI Score at the Lesion Level

The overall prevalence of malignancy per lesion at histology was 18.4% (277 of 1502), 11.5% (15 of 130) for nonadnexal masses and 19.1% (262 of 1372) for adnexal masses, including 45 (3.0%) borderline tumors. Detailed analysis of imaging criteria and their diagnostic performances are available in eTable 1 and eTable 2 in the Supplement.

The origin of each pelvic mass was correctly categorized as adnexal with a sensitivity of 0.99 (95% CI, 0.98-0.99; 1360 of 1372), a specificity of 0.78 (95% CI, 0.71-0.85; 102 of 130), a PLR of 4.60 (95% CI, 3.31-6.39), an NLR of 0.01 (95% CI, 0.01-0.02), a PPV of 0.98 (95% CI, 0.97-0.99), an NPV of 0.89 (95% CI, 0.82-0.94), and an accuracy of 0.97 (95% CI, 0.96-0.98). The diagnosis of the origin was reproducible with a substantial κ of 0.68 (95% CI, 0.59-0.76) between junior and experienced readers.

## Discussion

In this multicenter prospective cohort study, we demonstrated that a previously published^14^ 5-point MRI score provided robust risk stratification of sonographically indeterminate adnexal masses. The study confirms a strong concordance of the PLR of malignant neoplasms for each category. Therefore, the MRI score may provide potentially crucial information for determining the therapeutic strategy, allowing the risks and benefits of expectant management or surgery to be considered case by case.^19^ The study demonstrated the feasibility of the acquisition of the multiparametric MRI in multiple centers. Substantial interrater agreement was found, regardless of reader experience, which has been reported to be challenging in some ultrasonographic studies.^20,21,22^ External validations in smaller single-center studies have reported similar findings.^23,24,25^ The O-RADS MRI score is now proposed as the accepted score for risk assignment of sonographically indeterminate adnexal masses, supported by this strong evidence base.

The O-RADS MRI score addresses a significant clinical issue, given that approximately 18% to 31% of adnexal lesions detected on ultrasound remain indeterminate.^3,4,26,27,28,29^ Transvaginal sonography is accurate for detecting and characterizing adnexal lesions of classic appearance.^30^ However, in the 2 largest ovarian cancer screening trials,^7,8^ a significant number of false-positive cases underwent inappropriate surgery. Nonclassical features, such as avascular solid components, large masses, and less experienced sonographers could all contribute to lower accuracy and specificity on ultrasound examination.^20,31^ Several sonographic rules and scoring systems have been advocated, such as IOTA, Risk of Malignancy Index, and Risk of Ovarian Malignancy Algorithm.^3,10,11,27^ However, performance in real-life clinical settings has been variable, potentially because of differences in operator experience and cancer prevalence in the population being studied.^27,32,33,34^

Correctly classifying an adnexal mass as benign has positive consequences, including the potential to reduce overtreatment by unnecessary or overextensive surgery, to allow consideration of minimally invasive or fertility-preserving surgery, and to improve patient information regarding the risk of ovarian reserve alteration after surgery. The preponderant contribution of MRI in adnexal mass evaluation is its specificity, allowing confident diagnosis of many benign adnexal lesions.^19^ Using the O-RADS MRI score, our study demonstrated that, even in sonographically indeterminate masses, a lesion with a score of 2 has a PLR of malignant tumor of no greater than 0.01, and a lesion with a score of 3 has the PLR of malignant tumor of 0.27 among both experienced and junior readers. Thus, patients with lesions with scores of 2 or 3 can make an informed decision with the support of their physicians to undergo a minimally invasive or conservative surgical approach or expectant management. Such a high-performance clinical scoring system could allow for the development of decision-support tools, with referral of patients for appropriate follow-up vs surgery, and ensure that fertility-preserving treatment options are considered for young patients with early-stage disease.^35^ Our study showed that the likelihood of a borderline tumor when a lesion scores 5 was very low (<6%), as in a previous publication.^14^ However, as borderline tumors are a rare entity, our population included less than 3% (45 of 1502), and larger specific studies are needed.

Optimal management also relies on identifying the site of origin of a pelvic mass (ie, adnexal or nonadnexal). Our study showed that MRI helped to correctly reclassify the origin of the presumed adnexal mass on ultrasonography. In 802 women with only 1 mass described on MRI, 81 lesions (10.0%) were nonadnexal. This is particularly important for malignant nonadnexal tumors, for which initial incorrect management could adversely affect prognosis. In our population, 5.4% (15 of 277) of malignant tumors were nonadnexal lesions of uterine, colorectal, urothelial, nonepithelial peritoneal, or lymph node origin.

### Limitations

This study has limitations. It was observational and without randomization, and the score was not integrated into clinical decision-making. Therefore, the clinical consequences on the number of cases in which surgery can be avoided or tailored can only be imputed. However, the validation of the score now allows studies to test the consequences of the O-RADS MRI score in treatment planning; 2 such studies are currently underway.^36,37^ Furthermore, because patients were managed according to clinical recommendations, when no pelvic mass was found on MRI, no specific follow-up was undertaken in clinical care as in previous base studies.^4,5^ Consequently, 64 such cases were excluded from our analysis. This is a low proportion compared with the number of resolving lesions that are typically seen in general outpatient adnexal ultrasonography, given that most physiological ovarian masses are recognized and not referred for MRI. Thus, the O-RADS MRI score estimates the risk of malignancy of an existing pelvic mass detected on MRI. Magnetic resonance imaging is not recommended as a screening tool, and as such, the NPV when no mass is found is not available in the literature. In our study, 284 women had 2 lesions and 44 women had 3 lesions. As each mass was not considered independently, a potential clustering effect should be considered. In addition, 99 of 362 patients were observed during 2 years with only clinical assessment that cannot replace imaging evaluation. Furthermore, we did not include patients who were lost to follow-up in the final analysis. This could have biased the prevalence of the disease in the population, and that is why we calculated the PLR of malignant neoplasms and not the PPV. Of note, more than 90% of these patients were diagnosed with benign lesions on MRI, and this is likely to have played a part in the decision not to undertake further clinical follow-up.

## Conclusions

In conclusion, this prospective multicenter cohort study confirmed the performance of a 5-point scoring system developed in a previous retrospective single-center study. The current study provides strong supporting evidence, and the score is now presented as the O-RADS MRI score. Using this score in clinical practice may allow a tailored, patient-centered approach for masses that are sonographically indeterminate, preventing unnecessary surgery, less extensive surgery, or fertility preservation when appropriate, while ensuring preoperative detection of lesions with a high likelihood of malignancy.

## Notes

Supplement. **eAppendix 1. **Study Centers, Names of Principal Investigators, and Number of Patients Recruited and Included **eAppendix 2.** Trial Protocol **eAppendix 3. **Population Selection Criteria **eAppendix 4. **Methodological Details **eTable 1. **Lexicon and Supplementary Material with Detailed Analysis of Adnexal Lesions Based on Morphological Characteristics **eTable 2. **Diagnostic Values of Different MRI Features **eFigure. **Score Performance Among Senior and Junior Readers
