# Supplementary material for: Ovarian-Adnexal Reporting Data System Magnetic Resonance Imaging (O-RADS MRI) Score for Risk Stratification of Sonographically Indeterminate Adnexal Masses
Source: JAMA Netw Open. 2020 Jan 24;3(1):e1919896. doi: 10.1001/jamanetworkopen.2019.19896 (PMC6991280; doi:10.1001/jamanetworkopen.2019.19896)
Supplement: Supplement. — eAppendix 1. Study Centers, Names of Principal Investigators, and Number of Patients Recruited and Included eAppendix 2. Trial Protocol eAppendix 3. Population Selection Criteria eAppendix 4. Methodological Details eTable 1. Lexicon and Supplementary Material with Detailed Analysis of Adnexal Lesions Based on Morphological Characteristics eTable 2. Diagnostic Values of Different MRI Features eFigure. Score Performance Among Senior and Junior Readers [file jamanetwopen-3-e1919896-s001.pdf]

Supplementary Online Content

Thomassin-Naggara I, Poncelet E, Jalaguier-Coudray A, et al. Ovarian-Adnexal Reporting Data System Magnetic Resonance Imaging (O-RADS MRI) score for risk stratification of sonographically indeterminate adnexal masses. *JAMA Netw Open*. 2020;3(1):e1919896. doi:10.1001/jamanetworkopen.2019.19896

eAppendix 1. Study Centers, Names of Principal Investigators, and Number of Patients Recruited and Included
eAppendix 2. Trial Protocol
eAppendix 3. Population Selection Criteria
eAppendix 4. Methodological Details
eTable 1. Lexicon and Supplementary Material with Detailed Analysis of Adnexal Lesions Based on Morphological Characteristics
eTable 2. Diagnostic Values of Different MRI Features
eFigure. Score Performance Among Senior and Junior Readers

This supplementary material has been provided by the authors to give readers additional information about their work.

© 2020 Thomassin-Naggara I et al. *JAMA Network Open.*

eAppendix 1. Study Centers, Names of Principal Investigators, and Number of Patients Recruited and Included

Population (blue: inclusion / orange: final population) Clinical trial NCT01738789

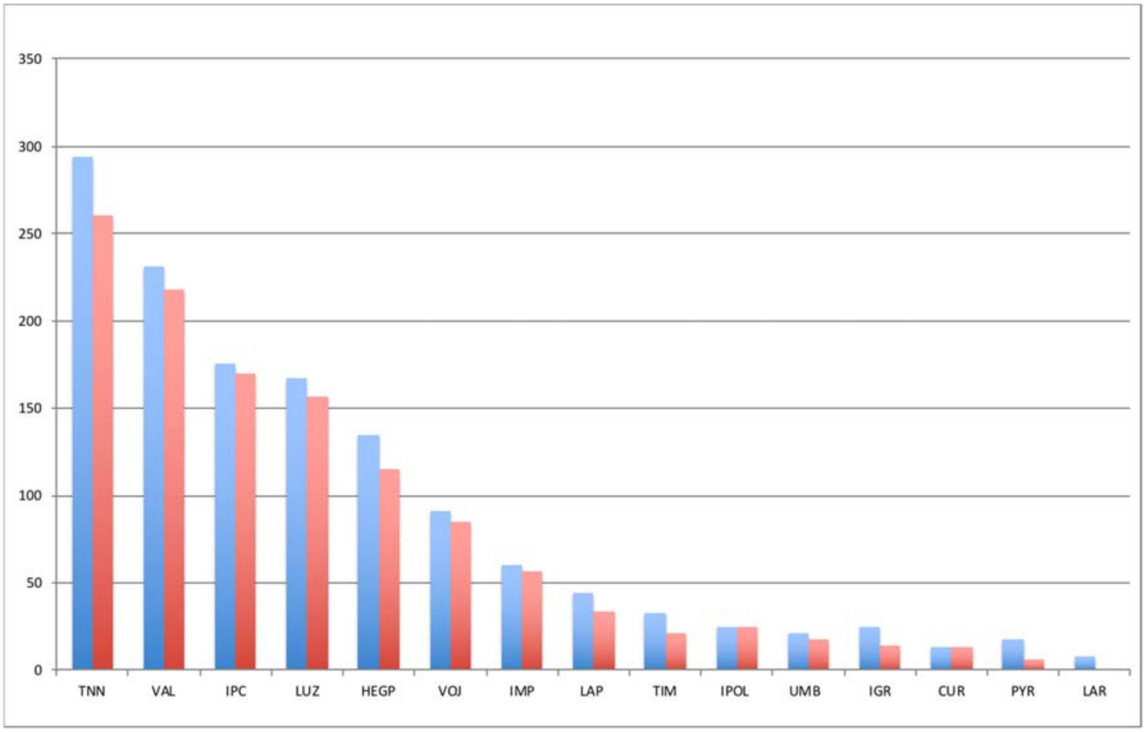

TNN: Hopital Tenon; VAL : Hopital de Valenciennes / IPC : Institut Paoli Calmettes, LUZ Hospital da Luz, HEGP Hopital Européen Georges Pompidou, VOJ : Clinical Centre of Vojvodine, IMP: Imperial College Healthcare NHS Trust, LAP : Hopital de Lapeyronie, TIM: Hopital de la Timone, IPOL: Instituto Português de Oncologia de Lisboa Francisco Gentil , UMB Umberto I hospital. Sapienza University (UHS) , IGR : Institut Gustave Roussy, CUR : Institut Curie, PYR : Centre Pyramides, LAR : Hopital Lariboisiere

© 2020 Thomassin-Naggara I et al. *JAMA Network Open.*

eAppendix 2. Trial Protocol

European multicenter validation of an ADNEXMR SCORING System for characterizing adnexal masses: "EURAD-MR classification"

**Title**	**European multicenter validation of an ADNEXMR SCORING System for characterizing adnexal masses: "EURAD-MR classification".**
**Short Title**	EURAD-MR classification: European multicenter study
**Version**	1.7
**Date**	01 decembre 2012
**Main investigator**	Dr I.Thomassin-Naggara
Speciality	Radiologist
Hospital Name	Hopital Tenon
Phone number	+33 1 56 01 64 53
e-mail	isabellethomassin@gmail.com
**CoMain investigator**	Pr A.Rockall
Speciality	Radiologist
Hospital Name	Imperial College Healthcare NHS Trust
Phone number	
e-mail	a.rockall@imperial.ac.uk
**CoMain investigator**	Pr M.Bazot
Speciality	Radiologist
Hospital Name	Hopital Tenon
Phone number	+33 1 56 01 64 53
e-mail	marc.bazot@tnn.aphp.fr

© 2020 Thomassin-Naggara I et al. *JAMA Network Open.*

TABLE

Section 1 : Abstract....4

Section 2 : Specific information CCTIRS....6

I - Nom, titres, expériences et fonctions de la personne responsable de la mise en oeuvre du traitement automatisé....6

II - Catégories de personnes....6

Section 3: Project description....7
Chapter 1 : Coordinator and particpating teams....7
Chapter 2 : Coordinator experience in the field....9
Chapter 3 : Clinical research project....10
3-1 Synopsis....10
3-1-1 Project description....10
3-1-2 Project feasibility....11
3-2-2 Planned schedule and key steps....12
3-2 Introduction....13
3-2-1 Pelvic MR imaging....13
3-3 Objectives....14
3-4 Experimental plan....15
3-4-1 Type of experimental plan....15
3-4-2 Study workflow....15
3-4-2-2 Data acquisition....15
3-4-2-3 Data analysis....16
3-4-2-3 Surgery or follow up....16
3-4-3 Expected duration of the research....17
3-3-4 Calendar....17
3-4-5 Criteria for cancellation....17
3-5 Evaluated parameters....17
3-5-1 Primary evaluation parameter....17
3-5-2 Secondary evaluation parameter....17
3-6 Safety evaluation....17
3-7 Statistics....18
3-7-1 External validation of ADNEX MR SCORING system....18
3-7-2 Evaluation of agreement between readers....18
3-8 Ethics and CRF....18
3-9 Data management....19
3-9-1 Data management....19
3-9-2 Right of access to data and source documents....19
3-10 Ethical and legal considerations....19
3-10-1 Request for the opinion of the CCP....19
3-10-2 Modifications....20
3-10-3 CCTIRS and Commission of Informatics and Freedom (CNIL) Declaration....20
3-10-4 Information letter and informed consent....20
3-10-5 Final research report....21
3-11 Rules concerning publication....21
3-12 References....21

© 2020 Thomassin-Naggara I et al. *JAMA Network Open.*

Appendix 1 : Lexicon* 23
Appendix 2 : AdnexMR scoring system (From Thomassin-Naggara et al. Radiology 2012 in press)........ 24
Appendix 3 : Informed form........ 25

Section 1 : Abstract

**Title of the project abstract**:
**INTRODUCTION**<br>An adnexal mass is the most common indication for gynaecological surgery [1]. Pre operative characterization is crucial and a scoring system would be useful to standardize the imaging report and thus, improve patient management. In the literature, various scoring systems have been developed based on clinical criteria, biochemical parameters (CA 125) and ultrasonographic criteria [2-3]. Recently, our center developed the first MR scoring system named ADNEXMR SCORING system in a retrospective study which is accurate (AUROC >0.94) and reproducible (K >0.846) in distinguishing benign from malignant masses [4]. Our primary objective is to perform an external prospective validation of the accuracy of this scoring system in a large multicenter study. Our secondary objectives are 1) to evaluate its potential impact on therapeutic strategy, in particular the possible reduction in inappropriate surgery in benign cases and 2) to test its reproducibility.<br><br>**EXPERIMENTAL PLAN**<br>This is a prospective multicenter study. All patients with a sonographically indeterminate adnexal mass referred for MR imaging will be consecutively included in each center. Then, patients will undergo a routine pelvic MR imaging (1.5T or 3T) in this setting, including: Morphological sequences (T2, T1 with and without fat suppression and T1 after dynamic gadolinium injection) and functional sequences (perfusion and diffusion-weighted sequences). Prospectively, one senior (expertise in pelvic MR imaging >5 years) and one junior radiologists (expertise in pelvic MR imaging 6-12months) independently analyze the different MR criteria to characterize adnexal masses. The MR report will be issued as standard and the patient will be managed accordingly. Then, the reader will classify the mass using ADNEXMR SCORING system. The classification will be compared to the reference standard as defined below.<br>The reproducibility of the classification will be tested between the junior and the senior radiologist. After anonymisation, images will be analyzed by another senior radiologist of another center blinded from any clinical or ultrasonographical data at the end of the study and correlated with the reference standard.<br><br>**Reference standard:** Reference standard will be surgical procedure with histology or standard clinical follow-up depending on most appropriate routine practice.<br><br>**Sample size:** The sample size was computed to ensure a power of at least 90% (with a two-sided type I error rate of 5%) to conclude that ADNEXMR SCORE 2 and 3 and ADNEXMR SCORE 4 and 5 would have a different PPV. It would thus be necessary to have at least 569 patients classified as ADNEXMR SCORE 2, 259 as ADNEXMR SCORE 3, 52 as ADNEXMR SCORE 4 and 51 as ADNEXMR SCORE 5 (18). Given the prevalences, and assuming 6% of patients would be classified, as ADNEXMR SCORE 1 and 10% would be lost to follow-up, **1340 patients** will be included in this study to insure a probability of at least 95% to obtain the aforementioned number of patients in each score category. The inclusion period will last 18 months and monitoring will continue.

© 2020 Thomassin-Naggara I et al. *JAMA Network Open.*

**Titre du projet**<br>**Résumé**:
**INTRODUCTION**<br>Les masses annexielles sont la première indication de chirurgie gynécologique [1]. La caractérisation pré opératoire est essentiel et une classification diagnostique a été développée pour standardizer les comptes rendus d'IRM pelvienne et améliorer la prise en charge des patients. Dans la literature de multiples scores existent incluant des critères cliniques, biologiques (CA 125 et échographiques [2-3]. Récemment, notre centre a développé le premier score en IRM pelvienne appelé ADNEXMR SCORING system dans une etude retrospective performant (AUROC >0.94) et reproductible (K >0.846) pour différencier les lesions bénignes et malignes [4]. Notre objectif principal est dans cette nouvelle etude de valider de facon externe et multicentrique ce score. Nos objectifs secondaires sont de tester sa reproductibilité de facon prospective et d'évaluer son potential impact thérapeutique.<br><br>**EXPERIMENTAL PLAN**<br>Il s'agit d'une étude prospective multicentrique. Toute patiente adressée en IRM pelvienne pour caractérisation d'une masse annexielle complexe en échographie sera incluse de facon consécutive dans chaque centre. Ainsi, les patientes effectueront un examen IRM selon le protocole de routine incluant des sequences pondérées T2, des sequences pondérées T1, des sequences de diffusion et de perfusion ainsi que des séquences classiques après injection de gadolinium. De facon prospective et indépendante, un radiologue senior (expertise >5ans) et un radiologue junior (expertise <1an) analyseront les différents critères IRM permettant d'effectuer une classification selon ADNEXMR SCORING system. Ce score sera confronté au Reference standard defini ci dessous. Pour évaluer la reproductibilité de la classification, une lecture sera réalisée par un radiologue senior et un radiologue junior. Après anonymisation, les images seront envoyées au centre coordonnateur de l'étude pour une analyse par un autre radiologue senior n 'ayant aucune donnée clinique ou échographique.<br><br>**Reference standard:** Le reference standard sera l'analyse anatomopathologique au décours de la prise en charge chirurgicale ou en l'absence de chirurgie (lesion typiquement bénigne) un suivi en imagerie selon les recommandations cliniques usuelles**.**<br><br>**Mille trois cent quarante patientes** doivent être incluses pour obtenir une probablilité d'au moins 95%. L'effectif a été calculé pour assurer une puissance d'au moins 90% (with a two-sided type I error rate of 5%). Pour conclure que ADNEXMR SCORE 2 and 3 and ADNEXMR SCORE 4 and 5 auraient des valeurs prédicitives positives différentes, il faudrait avoir au moins 569 patientes classées ADNEXMR SCORE 2, 259 classées ADNEXMR SCORE 3, 52 classées ADNEXMR SCORE 4 and 51 classées ADNEXMR SCORE 5 (18). Compte tenu des prévalences, considérant que 6% des patientes seront classées ADNEXMR SCORE 1 et que 10% des patientes seront perdues de vue, la période d'inclusion sera de 18 mois avec un suivi en imagerie.

© 2020 Thomassin-Naggara I et al. *JAMA Network Open.*

Section 2 : Specific information CCTIRS

I - Nom, titres, expériences et fonctions de la personne responsable de la mise en oeuvre du traitement automatisé

Isabelle Thomassin-Naggara
Maitre de Conférences Universitaires- Praticien Hospitalier
Investigateur coordonnateur

II - Catégories de personnes :

. qui seront appelées à mettre en oeuvre le traitement automatisé des données :
Investigateur principal dans chaque site

. qui auront accès aux données :
Investigateur coordonnateur
Assistant de recherche clinique du centre coordonateur

III – La recherche fait-elle appel aux données du SNIRAM ?

Si oui, il faut l'indiquer dans le protocole de recherche (en justifiant de leur utilisation). Dans ce cas, l'avis favorable du comité dispense de soumettre à l'Institut des données de santé chargé de contrôler cet accès.

Non

© 2020 Thomassin-Naggara I et al. *JAMA Network Open.*

Section 3: Project description

Chapter 1 : Coordinator and particpating teams

List of participating teams *Add as many lines as necessary*					
N°		Name of the team/laboratory or hospital department	Affiliated institution and city	Title, name of the team manager in the frame of the project	Contact email
1		Institut Paoli-Calmettes (IPC)	Marseille, FR	Dr A.Jalaguier	aureliejalaguier@yahoo.fr
2		Centre Oscar Lambret (COL)	Lille, FR	Dr S.Taieb	S-Taieb@o-lambret.fr
3		Institut Gustave Roussy (IGR)	Villejuif, FR	Dr C.Balleyguier	balleyguier@igr.fr
4		Hôpital de la Pitié Salpétrière (SAL)	Paris, FR	Pr O.Lucidarme	olivier.lucidarme@gmail.com
5		Hôpital Européen Georges Pompidou (HEGP)	Paris, FR	Dr L.Fournier	laure.fournier@gmail.com
6		Hopital Lapeyronie (LAP)	Montpellier, FR	Dr I.Millet	patricetaourel@wanadoo.fr
7		Centre imagerie pyramides (CIP)	Paris, FR	Dr N.Perrot	vitenson@wanadoo.fr
8		Hopital Lariboisière (LAR)	Paris, FR	Dr S.Bendavid	sandrabendavid@free.fr
9		Hopital de Valenciennes (VAL)	Valenciennes, FR	Dr E.Poncelet	poncelet.edouard@gmail.com
10		Hopital de la Timone (TIM)	Marseille, FR	Dr V.Juhan	Valerie.JUHAN@ap-hm.fr
11		Institut Curie (CUR)	Paris, FR	Dr E.Aubert	emi.aubert@yahoo.fr
12		Hôpital Tenon (Coordinator) (TNN)	Paris, FR	Dr I.Thomassin-Naggara	isabellethomassin@gmail.com
13		Imperial College Healthcare NHS Trust (ICH)	London, UK	Pr A.Rockall Dr N.Bharwani	a.rockall@imperial.ac.uk
14		Barts Health NHS	London, UK	Dr Anju	Anju.Sahdev@bartshealth.nhs.uk

© 2020 Thomassin-Naggara I et al. *JAMA Network Open.*

		Trust (BAR)		Sahdev	
15		Steeping Hill hospital	StockPort, UK	Dr M.Lewinski	Maryna.Lewinski@stockport.nhs.uk
16		Addenbrookes hospital (ADD)	Cambridge, UK	Dr S.Freeman	
17		University College Hospital London	London UK	Prof M Hall-Craggs	Margaret.hall-Craggs@uclh.nhs.uk
18		University Hodpital Dubrav	Bonn, SW	Dr R.Kubik	Rahel.Kubik@ksb.ch
19		John Paul Catholic University (JPCU)	Campobasso, IT	Pr G.Reistano	grestaino@rm.unicatt.it
20		Umberto I hospital. Sapienza University (UHS)	Roma, IT	Dr G.Masseli	g.masselli@policlinicoumberto1.it
21		Instituto di Radiologia	Verone, IT	Pr R.Manfredi/Pr R.Pozzi Mucelli	roberto.pozzimucelli@uniivr.it
22		Clinique des Grangettes	Geneve, SW	Pr K.Kinkel	karen.kinkel-trugli@wanadoo.fr
23		University Institute of Radiology (UIR)	Salzburg, AUS	Pr R Forstner	R.Forstner@salk.at
24		Instituto Português de Oncologia de Lisboa Francisco Gentil (IPOLFG)	Lisboa, PT	Pr T.M. Cuhna Dr C.Campos	tmargarida@gmail.com
25		Hospital da Luz (LUZ)	Lisboa, PT	Dr Gisa Guerra	gisaguerra@gmail.com
26		Clinical Center of Vojvodine	Novi Sad, SER	Dr S.Stojanovic	tupsons@gmail.com
27		CHU liège	Liege, BE	Dr A.Thille	alain@thille.be
28		University Hodpital Dubrav	Zagreb,CR	Dr I.Gordana	gordana.augustan@gmail.com

© 2020 Thomassin-Naggara I et al. *JAMA Network Open.*

Chapter 2 : Coordinator experience in the field

Principal significant articles published over the last five years
1- Thomassin-Naggara I., et al. Development and preliminary validation of an MRI Scoring system for Adnexal Masses. **Radiology** in press, accepted on October, 14rd 2012
2- Thomassin-Naggara I., et al. Quantitative analysis of dynamic contrast enhanced MR imaging of complex adnexal masses: A preliminary study. **Eur Radiol**, 2012, Apr, 22(4) : 738-453. Original Article
3- Thomassin-Naggara, I., et al., Characterization of Complex Adnexal Masses: Value of Adding Perfusion- and Diffusion-weighted MR Imaging to Conventional MR Imaging. **Radiology**, 2011. 258(3): p. 793-803.
4- Thomassin-Naggara, I., et al., Contribution of diffusion-weighted MR imaging for predicting benignity of complex adnexal masses. **Eur Radiol**, 2009. 19(6): p. 1544-52.
5- Thomassin-Naggara, I., et al., Dynamic Contrast-Enhanced Magnetic Resonance Imaging: a Useful Tool for Characterizing Ovarian Epithelial Tumors. **J Magn Reson Imaging**, 2008. 25(1): p. 111-120.
6- Thomassin-Naggara, I et al. Epithelial ovarian tumors: Value of dynamic contrast enhanced MR imaging and correlation with tumor angiogenesis. **Radiology**. 2008 Jul;248(1):148-59. Epub 2008 May 5.
7- Thomassin-Naggara, I et al. Dynamic contrast enhancement of ovarian neoplasm: current status and future perspectives. **Magn Reson Imaging Clin N Am**. 2008 Nov; 16(4): 661-72, ix. Review.
8- Thomassin-Naggara, I et al. Epithelial ovarian tumors: Value of dynamic contrast enhanced MR imaging and correlation with tumor angiogenesis. **Radiology**. 2008 Jul;248(1):148-59. Epub 2008 May 5.

© 2020 Thomassin-Naggara I et al. *JAMA Network Open.*

Chapter 3 : Clinical research project

3-1 Synopsis

3-1-1 Project description

**Justification and interest of the study**
Ovarian cancer is the fourth leading cause of death among neoplastic diseases in women. The unfavourable prognosis is due to the fact that 75% of cases are diagnosed at an advanced stage (FIGO stage III and IV) because of the non-specific nature or total absence of symptoms in the initial phase of the disease. This data probably explains why there is still a widespread tendency among surgeons to remove most of sonographically indeterminate adnexal masses despite of a low rate of malignancy (<20%)[1]. Consequently, a significant number of women with such masses unnecessarily undergo cancer surgery with consequences relating to morbidity and fertility in young women. Moreover, other women with suspected ovarian cancer may not be systematically referred to a cancer center with a specialised gynaecological oncologist and thus do not undergo an appropriate first surgical procedure. This type of suboptimal management includes a substantial risk of incomplete resection after the first surgery, which is one of the most important prognostic factors in ovarian cancer. Therefore, pre operative characterization is crucial and a scoring system would be useful to standardize imaging reports and thus, improve patient management. In the literature, various scoring systems have been developed based on clinical criteria, biochemical parameters (CA 125) and ultrasonographic criteria [2-3]. Most of them are not easy to apply in clinical routine, and, to date, none is considered as a suitable preoperative diagnostic method. Recently, our center developed an MR scoring system named ADNEXMR SCORING system in a retrospective study including 497 indeterminate adnexal masses in two years [4]. This classification was inspired by the BI-RADS classification and contains five categories according to the positive likelihood ratio for malignancy of significant MR features. Our study demonstrated that the classification is accurate (AUROC >0.94), reproducible (K >0.846) and readily distinguishes benign from malignant masses.

**Main orientations of the project**<br>If this project has not been previously selected (case 2), mention the additional aspects relevant to the recommendations of the Scientific Committee.
**Objectives of the clinical research**<br>• **Primary objective:**<br>Evaluate if ADNEXMR SCORING system is accurate for reporting pelvic magnetic resonance imaging (MRI) examinations performed for characterization of sonographically indeterminate adnexal masses<br>• **Secondary objectives:**<br>Evaluate<br>a) The potential impact of applying the score to the therapeutic strategy, in particular to measure the possible reduction in inappropriate surgery in benign cases

© 2020 Thomassin-Naggara I et al. *JAMA Network Open.*

b) If ADNEXMR SCORING system improves reproducibility of MR report for characterization of adnexal masses
c) If ADNEXMR SCORING system is as accurate if the radiologist is blinded from any clinical and ultrasonographic data

Main endpoint
Joint analysis of true negative and false negative rates according to ADNEXMR SCORING system as compared to the histological results (or follow-up outcome, see "reference standard", below)

Experimental plan
A prospective multicenter study (phase III diagnostic study according to the classification of Gluud & Gluud). (1) All patients with a sonographically indeterminate adnexal mass referred for MR imaging will be consecutively included in each center. Then, patients will undergo a routine pelvic MR imaging (1.5T or 3T) in this setting, including: Morphological sequences (T2, T1 with and without fat suppression and T1 after gadolinium injection) and functional sequences (perfusion and diffusion-weighted sequences). Prospectively, one senior (expertise in pelvic MR imaging >5 years) and one junior radiologists (expertise in pelvic MR imaging 6-12monthes) analyze the different MR criteria to characterize adnexal masses. The MR report by the senior radiologist will be issued as standard and the patient will be managed accordingly. Then, the reader will classify the mass using ADNEXMR SCORING system. The classification will be compared to the Reference standard as defined bellow.
To evaluate the reproducibility of the classification, after anonymisation, images will be analyzed by another senior radiologist from another center at the end of the study and correlated with the reference standard.

Reference standard
Routine clinical management which can include surgical procedure and histology or standard clinical follow-up depending on most appropriate routine practice.

3-1-2 Project feasibility

**Inclusions**	
**Number of required patients**	**Number of required patients: 1340**<br>The sample size was determined based on results of the development and first validation study. Overall, because of the preliminary validation, we may expect that slightly less than 5% of patients would be classified as ADNEXMR SCORE 1; these patients will not be part of the validation testing. Among remaining patients the expected prevalence of each category and associated probability of events are summarized hereunder:

ADNEX MRSCORE	Probablility of prevalence	P (Cancer) (PPV)	OR	OR*
2	0.55	0.01	1	-
3	0.25	0.05	5.21	-
4	0.10	0.50	99	1
5	0.10	0.80	396	4.0

Given this table, the sample size was computed to ensure a power of at least 90% (with a two-sided type I error rate of 5%) to conclude that ADNEXMR SCORE 2 and 3 and ADNEXMR SCORE 4 and 5 would have a different PPV. It would thus be necessary to have at least 569 patients classified as ADNEXMR SCORE 2, 259 as ADNEXMR SCORE 3, 52 as ADNEXMR SCORE 4 and 51 as ADNEXMR SCORE 5 (18). Given the prevalences, and assuming 6% of patients

© 2020 Thomassin-Naggara I et al. *JAMA Network Open.*

	would be classified, as AdnexMR score 1 and 10% would be lost to follow-up, it is necessary to include a total number of **1340** patients to insure a probability of at least 95% to obtain the aforementioned number of patients evaluated.
**Number of centers for patients accrual**	28 centers
**Feasibility**<br>All patients addressed to the 28 study centers that fulfill the selection criteria are included.<br>The average number of available patients per year is 570 patients in France and 320 in other European countries. These numbers are based on the report of pelvic MRI for characterization of indeterminate adnexal masses at the radiology department in the different hospitals	

3-2-2 Planned schedule and key steps

Estimated schedule and identification of key steps
**Inclusion:** 18 months<br>**Follow up after inclusion:** 24 months<br>**Total:** 3.5 years
**First inclusion**: 1 January 2013<br>**End of the inclusions**: 30 June 2014<br>**Follow up**: from 1 July 2014 to 31 June 2016<br>**Data analysis**: July 2016

© 2020 Thomassin-Naggara I et al. *JAMA Network Open.*

3-2 Introduction

Preoperative characterization of indeterminate adnexal masses is crucial and a scoring system should be useful to standardize the imaging report and thus, improve patient management. Ultrasonography is the first-line imaging technique for detecting and characterizing ovarian tumors [5]. In this specific setting, several authors have proposed various scores taking into account epidemiological characteristics, serum tumor marker levels and ultrasonographic features, including the presence of solid tissue [2]. Nevertheless, in a prospective randomized trial, Yazbek et al found that the diagnostic accuracy of ultrasonography in this setting was operator dependent [6]. Moreover, certain conditions that hinder accurate transvaginal examination, such as lesion complexity, large tumor size, obesity and virginity, are indications for MR imaging, which is superior to CT for assessing indeterminate ovarian tumors [7, 8].

3-2-1 Pelvic MR imaging

MR imaging was proven to be the most accurate imaging technique and the most cost effective intervention for ultrasonographically indeterminate masses: Firstly, the risk of malignancy for these masses is small (lower than 20% of malignancy), particularly in premenopausal women. Secondly, the intervention of MR imaging results in improved surgical treatment. Thirdly, by removing the need for follow up US, MR imaging offers the possibility of earlier diagnosis of cancer in that minority of women with malignant masses [7]. In the literature, the accuracy of MRI for distinguishing malignant from benign indeterminate adnexal masses ranges from 83% to 93% [9-11], compared to 63%-80% with ultrasonography [10]. Recently, functional imaging techniques adding new criteria to conventional MRI improve the interpretation, reproducibility and characterization of indeterminate adnexal masses, resulting in an accuracy higher than 95.3% [12].

3-2-2 Development of an MR scoring system

ADNEXMR SCORING system is the first MR scoring system based on MR features. The features included in our MR scoring system are well established, as each has previously been used to distinguish benign from malignant masses [9, 11-17]. Our classification develops ADNEXMR SCORE 2 where no further investigations are necessary based on five MR features: purely cystic, purely endometriotic mass, purely fatty mass, low T2 and b1000-weighted signal within solid component and no cyst wall enhancement. All of the mentioned criteria are well known to be useful in the description of indeterminate adnexal masses, except the absence of wall enhancement, which is new and should be validated prospectively. The limitations of the study, which developed ADNEXMR SCORING system, was the low number of borderline tumors (n=6) and the retrospective single center design of the study.

Thus, the value and the reproducibility of this scoring system need to be confirmed in a larger prospective multicenter study.

3-2-3 Potential significance of a MR scoring system

© 2020 Thomassin-Naggara I et al. *JAMA Network Open.*

In our preliminary study, AdnexMR scoring system had good discriminatory characteristics with an AUROC of 0.981/0.964 for an expert reader and 0.961/0.943 for a junior reader in both the training and validating set. The reproducibility of the AdnexMR scoring system was almost perfect with an agreement of 0.848 for the training and of 0.888 for the validating set between the two readers. A preliminary external validation was performed on the validating set with 4 readers (2 senior and 2 juniors) that suggests a high diagnostic performance (AUROC 980, 0.954, 0.955, 0.973 for S1, S2, J1 and J2 respectively). For these four subsequent radiologists, the observed malignancy rates were less than 2 %, less than 5%, between 5% and 90% (precisely 37% (7/19), 40% (6/15), 50% (6/12) and 58% (7/12) for SD, PS, VJ and AJ) and over 90 % for AdnexMR-score 2,3,4 and 5, respectively. Therefore, this classification should be easily adopted by radiologists whatever their degree of expertise in pelvic imaging to improve report standardization.

Finally, AdnexMR scoring system should influence pelvic mass management. With a score 4 or 5, AdnexMR scoring system predicts malignancy with 93.5% of sensitivity and 96.6% of specificity. Thus, the risk of malignancy is high, and the patient should be referred to a cancer center to undergo an appropriate surgical procedure at the first attempt. With a score ≤ 3, the risk of malignancy is minimal and the patient may benefit from more imaging follow-up or conservative treatment.

3-3 Objectives

- **Primary objective:**

Evaluate if AdnexMR scoring system is relevant for reporting pelvic magnetic resonance imaging (MRI) examinations performed for characterization of sonographically indeterminate adnexal masses in an external prospective multicenter study

- **Secondary objectives and endpoints:**

Evaluate

a) The potential impact of applying the score to the therapeutic strategy, in particular to measure the possible reduction of unnecessary surgery in benign cases
b) If AdnexMR scoring system improves reproducibility of MR report for characterization of adnexal masses
c) If AdnexMR scoring system is as accurate if the radiologist is blinded from any clinical and ultrasonographic data

Main endpoint

Joint analysis of true negative and false negative rates according to AdnexMR scoring system as compared to the histological results (or follow-up outcome, see "reference standard", below) with an evaluation of the sensitivity and the specificity of the score

3-4 Experimental plan

3-4-1 Type of experimental plan

A prospective multicenter study (phase III diagnostic study according to the classification of Gluud & Gluud).

3-4-2 Study workflow

The workflow will be the following, consisting of 3 phases

- Inclusion of patients
- Pelvic MR imaging
- Surgery or clinical follow-up

Patient management will be guided by routine clinical criteria and will be based on the recognized standards of care for patients of more than 18 years with a pelvic MR imaging abnormality. ADNEXMR SCORING system is the only thing added in this research project and the score will be analyzed separately from clinical care of the patient. The score will not given to clinician before the end of the study in order to not impact on the usual patient management.

All patients with a sonographically indeterminate adnexal mass referred for MR imaging will be consecutively included in each center.

Inclusion Criteria
- Patient ≥ 18 years old
- With sonographically indeterminate adnexal mass
- Informed consent

Exclusion Criteria
- Pregnant women (relative contra indication for gadolinium injection)
- Pacemaker, ferromagnetic materials, or foreign body at risk of mobilization or any other contra-indication to MR imaging.
- Intolerance to gadolinium contrast agents, or severe renal insufficiency (GFR <30 ml/min/1.73m²).

3-4-2-2 Data acquisition

Inclusion will be performed by the senior radiologist (or a delegated member of the team) to ensure each patient satisfies the inclusion criteria. Each patient will be informed of the study, will be provided with a patient information sheet and will be asked to give written informed consent to participate. An email will be sent to the main investigator at each inclusion.
Then, patients will undergo a routine pelvic MR imaging (1.5T or 3T) in this setting, including: Morphological sequences (T2, T1 with and without fat suppression and T1 after gadolinium injection) and functional sequences (perfusion and diffusion-weighted sequences) for every patient. If at the time of inclusion and MRI scan, the adnexal mass has disappeared on T2 and T1 weighted sequences, functional sequences and

© 2020 Thomassin-Naggara I et al. *JAMA Network Open.*

gadolinium injection will not be necessarily be performed. The patient initially included will be subsequently excluded and not considered in the number of the cohort of the center considered.
The first patient of each center will be sent to main investigator to check if acquisition protocol is correct.

3-4-2-3 Data analysis

Preliminary period : A session of 30 DICOM cases will be downloaded for a session for all teams participating to the multicenter validation (I.Thomassin-Naggara (GE) / A.Rockall (Siemens / Philips)

Prospective data analysis : The readers will be informed of clinical and ultrasonographic data. The quality of US report will be evaluated. Then, the readers will analyze MR imaging : In a first step, one senior (expertise in MR imaging >5 years) and one junior radiologists (expertise in MR imaging 6-12months) analyze the following criteria (Annexe 1): bilaterality, size, wall enhancement, bi or multilocularity, the presence of thickened regular septa or grouped septa. These criteria determine the presence of purely cystic, purely endometriotic and purely fatty mass (Cf lexicon). The presence of a solid component and its presentation (solid portion, vegetation, thickened irregular septa) will be evaluated. Then, T2-weighted signal intensity within the solid component (low or intermediate compared to the outer myometrium) and diffusion-weighted signal intensity within the solid component (high diffusion-weighted signal intensity compared to serous fluid; i.e. urine within bladder or cerebro-spinal fluid [CSF]) will be analyzed. The reader will classify the enhancement of the solid component using time intensity curve classification [15]. Finally, the presence of associated ascites and peritoneal implants will be also noted. Following interpretation of the above findings, the senior reader will issue a standard clinical report.

Then, the readers will classify the mass using ADNEXMR SCORING system (See below). The classification will be compared to the Reference standard as defined below.

If multiple adnexal masses are discovered during MR imaging, all lesions will be analyzed following the previous criteria detailed above. The lesion named “one” will be the lesion for which MR imaging was performed.

At the end of the procedure, DICOM data will be sent to the center coordinator (Hopital Tenon). At the end of the study, anonymized data will be posted on a securized web network. Another reading of each case will be performed by two other senior radiologists without any knowledge of clinical, ultrasonographic, pathological or follow up data. CRF pages will be sent by postal way to center coordinnator.

3-4-2-3 Surgery or follow up

If the lesion is considered as potentially malignant by the multidisciplinary team, surgery will be performed. The type and the interval between MRI scan and the date of surgery will be considered for the analysis.

If the lesion is considered benign, follow-up or surgery will be according to standard clinical practice. This could include clinical evaluation, ultrasound or further MRI. The final diagnosis of the patient at 24 months will be recorded. The diagnosis will be categorized as benign, borderline or malignant.

© 2020 Thomassin-Naggara I et al. *JAMA Network Open.*

3-4-3 Expected duration of the research

Recruitment will take place over 18 months and the follow-up time will be two years after finding a lesion considered as benign.
The total duration of the research is 3.5 years.

3-3-4 Calendar

First inclusion: 1 January 2013
End of the inclusions: 30 june 2014
Follow up: from 1 July 2014 to 30 june 2016
Data analysis: July 2016

3-4-5 Criteria for cancellation

The patient retains her right to withdraw from the study at any moment.

3-5 Evaluated parameters

3-5-1 Primary evaluation parameter

The primary evaluation parameter will be the classification error of tumors by the senior and by the junior according to the ADNEXMR SCORING system (divided into categories 4 and 5 versus 1 to 3) obtained by pelvic MR imaging according to the reference standard. Reference standard will be surgical procedure or clinical follow-up depending on a routine practice analysis. If the lesion is considered benign, the clinical and/or radiological diagnosis at 24 months will be recorded as the final diagnosis.

3-5-2 Secondary evaluation parameter

The reproducibility of the interpretation of the ADNEX MR SCORING system will be assessed with differences of ADNEX MR SCORING system observed between the junior and the senior radiologist propectively in a same center. Subsequently, we will test between two senior radiologists from two different centers. Thus, after anonymisation, images will be sent and analyzed by another senior radiologist of another center blinded from any clinical and ultrasonographical data at the end of the study and correlated with Reference standard.

The improvement of reproducibility will be given by the comparison with the reproducibility of routine conclusion given at the end of the report

3-6 Safety evaluation

The research protocol requires injection of contrast during pelvic MRI. Risks for patients undergoing MRI examinations are related to the contrast agent (gadolinium chelate). They include allergy (0.001%) and nephrogenic systemic fibrosis in case of kidney

© 2020 Thomassin-Naggara I et al. *JAMA Network Open.*

failure (clearance < 30 ml/min). In case of previous allergy to gadolinium chelate or kidney failure, no MRI will be performed and the patient will be excluded.

3-7 Statistics

3-7-1 External validation of ADNEX MR SCORING system

The sample size was determined based on results of the development and first validation study. Overall, we may expect that slightly less than 5% of patients would be classified as ADNEXMR SCORE 1; these patients will not be part of the validation testing. Among remaining patients the expected prevalence of each category and associated probability of events are summarized hereunder:

ADNEX MRSCORE	Probablility of prevalence	P (Cancer) (PPV)	OR	OR*
2	0.55	0.01	1	-
3	0.25	0.05	5.21	-
4	0.10	0.50	99	1
5	0.10	0.80	396	4.0

Given this table, the sample size was computed to ensure a power of at least 90% (with a two-sided type I error rate of 5%) to conclude that ADNEXMR SCORE 2 and 3 and ADNEXMR SCORE 4 and 5 would have a different PPV.
It would thus be necessary to have at least 569 patients classified as ADNEXMR SCORE 2, 259 as ADNEXMR SCORE 3, 52 as ADNEXMR SCORE 4 and 51 as ADNEXMR SCORE 5 (18). Given the prevalences, and assuming 6% of patients would be classified, as ADNEXMR SCORE 1 and 10% would be lost to follow-up, it is necessary to include a total number of **1340** patients to insure a probability of at least 95% to obtain the aforementioned number of patients evaluated.

3-7-2 Evaluation of agreement between readers

The agreement between the ADNEXMR SCORE for the two readers will be evaluated with the intraclass correlation (ICC). For ordinal scales such as ADNEXMR SCORE, ICC is equivalent to kappa statistics according to Cohen. Results will be presented with a 95% confidence interval.

3-8 Ethics and CRF

A registration number for the research will be obtained. The research protocol and the patient information sheet (PIS) will be presented to the Person Protection committee (CPP - Ile de France V).

A clinical study Technician (CST) will be recruited to assist the project in compliance with the Protocol and its circuits as well as filling of CRF in the recruitment centers.

© 2020 Thomassin-Naggara I et al. *JAMA Network Open.*

All information required by the protocol will be provided in the CRF and in the case of missing data, the investigator will give an explanation.
The data must be recorded in the CRF as soon as they are obtained both for clinical and non-clinical data.

3-9 Data management

3-9-1 Data management

For each patient all items defined in chapter "Evaluated parameters" will be filled out by the investigator radiologists. A CRF will be created to collect all data. Patient information will be given as following :
- SITE : Abbreviate name of the center (given in table page 5)
- PATIENT NUMBER : From 1 for each center
- INITIALS of first name and last name
- INCLUSION DATE

Documents under the law on biomedical research must be archived during 15 years after the end of research.
This indexed archive includes:
· Copies of the letter of the mandatory opinion of CPP
· Successive versions of the protocol (identified by version number and date)
· Signed consent forms in sealed storage with a list or register of enrollments
· CRF completed and validated for each patient
· All the appendices specified in the study
· The final study report, statistical analysis and quality control for the study
· Audit certificates from any audits done during the research
The database for the statistical analysis should also be archived by the person responsible of the analysis (paper or computer).

3-9-2 Right of access to data and source documents

Persons with direct access of data and source documents in accordance with the laws, including articles L.1121-3 and R.5121-13 of the French Public Health Low (code de santé publique) take all necessary precautions to ensure the confidentiality of information relating to experimental drug trials, testing, and persons involved, particularly regarding their identity and the obtained results. The data collected by these persons during quality control or audits will be anonymized.

3-10 Ethical and legal considerations

Patients will be informed of the execution of the protocol in a detailed information leaflet.
The investigator will collect the informed consent form prior to the radiology examination. The patient retains the right to withdraw their consent at any time, according to the law.

3-10-1 Request for the opinion of the CCP

© 2020 Thomassin-Naggara I et al. *JAMA Network Open.*

The research protocol must be sent to a CCP. The opinion of this committee is given to the competent authority by the sponsor before to start of the study. According to ECRIN recommendation, one referent by country will be named to send the protocol to one's CPP.

3-10-2 Modifications

During the whole project, modifications must be qualified as substantial or not.
A substantial modification is a change that modifies the warranties given to patients consenting to biomedical research (modification of inclusion criteria, extension of length of enrollment, participation of new centers…). After the first inclusion, any substantial modification must obtain, prior to its implementation, an agreement from the committee and authorization from the competent authority. In that case, if necessary, the committee will ensure that a new consent from persons participating in the research is used.
Otherwise, all extension of research (substantial modification to therapeutic workflow or to the included population, extension of treatment and/or treatment not initially foreseen in the protocol) must be considered as a new research project.
Any substantial modification will require an application to review by CPP.

3-10-3 CCTIRS and Commission of Informatics and Freedom (CNIL) Declaration

The law envisions that notification of computerized files of personal data must be done before the start of the research.
A specific reference method for handling personal data made in biomedical research as defined by the law 2004-806 from Aug 9, 2004 as falling within the scope of articles L.1121-1 of the French public health Law (Code de Santé Publique) was established by CNIL in January 2006.
This methodology permits a simplified notification procedure if the nature of the data collected in research is in accordance with the list provided by CNIL in their reference document.
The person responsible for the data files to give a written statement on compliance with the methodology of reference MR06001 simplified.

3-10-4 Information letter and informed consent

During the enrollment period, all patients who will undergo pelvic MR imaging for the characterization of a complex adnexal mass will meet an investigator radiologist (or a delegated member of the research team). This person will verify the inclusion/exclusion criteria of the patient and propose participation in the study, explaining clearly the objective of the study and what will happen. They will give an information letter to the patient and the consent form (triple copies) signed and dated by the investigator radiologist at the examination. If the patient agrees to participate, she will sign the consent form on the day of the examination with the study radiologist (or a delegated member of the research team), before doing the pelvic MR imaging according to protocol. The study radiologist will keep two dated and signed forms, and the patient will keep the third.

© 2020 Thomassin-Naggara I et al. *JAMA Network Open.*

The consent form with the name of the patient must be verified the same day. The original will be kept by the principal investigator in the study archives (archived for 15 years). The third copy will be sent to the principal investigator at the end of the study in a sealed envelope.
A separate register will cross-reference the patient number in the study with their name and hospital number. This list will be kept by the principal investigator in the archive.

3-10-5 Final research report

The final research report will be written by the coordinator in collaboration with the biostatistician. This report will be sent to all investigator radiologists for comments. Once consensus has been reached, the final version will be endorsed by the signature of each study radiologist and submitted to the sponsor as soon as possible after the effective end of the research. A report edited according to the reference plan of the competent authority must be sent to the competent authority and to CPP within a year of the end of the study, defined as the end of the follow-up for the last patient. This delay is 90 days in case of premature termination of the research.

3-11 Rules concerning publication

Scientific publications will be the responsibility of the principal investigator. The participation of centers in publications will follow two rules: number of enrollments in the study and proposal by the investigators involved in a supplementary publications. To be published in one of the journals of the International Committee of Medical Journal Editors (ICMJE, see http://www.icmje.org/jrnlist.html). Clinical trials should be listed, prior to enrollment of the first patient, at the website http://www.clinicaltrials.gov/. Only that site by the FDA/NIH corresponds today to the requirements of the editors of ICMJE.

3-12 References

1. Curtin, J.P., Management of the adnexal mass. Gynecol Oncol, 1994. 55(3 Pt 2): p. S42-6.
2. Timmerman, D., et al., Logistic regression model to distinguish between the benign and malignant adnexal mass before surgery: a multicenter study by the International Ovarian Tumor Analysis Group. J Clin Oncol, 2005. 23(34): p. 8794-801.
3. Brown, D.L., et al., Benign and malignant ovarian masses: selection of the most discriminating gray-scale and Doppler sonographic features. Radiology, 1998. 208(1): p. 103-10.
4. Thomassin-Naggara I., et al. Development and preliminary validation of an MRI Scoring system for Adnexal Masses. Radiology in press, accepted on 2012, October, 14rd
5. Gynecologic sonography. Report of the ultrasonography task force. Council on Scientific Affairs, American Medical Association. Jama, 1991. 265(21): p. 2851-5.
6. Yazbek, J., et al., Effect of quality of gynaecological ultrasonography on management of patients with suspected ovarian cancer: a randomised controlled trial. Lancet Oncol, 2008. 9(2): p. 124-31.

© 2020 Thomassin-Naggara I et al. *JAMA Network Open.*

7. Kinkel, K., et al., Indeterminate ovarian mass at US: incremental value of second imaging test for characterization--meta-analysis and Bayesian analysis. Radiology, 2005. 236(1): p. 85-94.
8. Tsili, A.C., et al., Comparative evaluation of multidetector CT and MR imaging in the differentiation of adnexal masses. Eur Radiol, 2008. 18(5): p. 1049-57.
9. Hricak, H., et al., Complex adnexal masses: detection and characterization with MR imaging-- multivariate analysis. Radiology, 2000. 214(1): p. 39-46.
10. Rieber, A., et al., Preoperative diagnosis of ovarian tumors with MR imaging: comparison with transvaginal sonography, positron emission tomography, and histologic findings. AJR Am J Roentgenol, 2001. 177(1): p. 123-9.
11. Sohaib, S.A., et al., Characterization of adnexal mass lesions on MR imaging. AJR Am J Roentgenol, 2003. 180(5): p. 1297-304.
12. Thomassin-Naggara, I., et al., Characterization of Complex Adnexal Masses: Value of Adding Perfusion- and Diffusion-weighted MR Imaging to Conventional MR Imaging. Radiology, 2011. 258(3): p. 793-803.
13. Siegelman, E.S. and E.K. Outwater, Tissue characterization in the female pelvis by means of MR imaging. Radiology, 1999. 212(1): p. 5-18.
14. Thomassin-Naggara, I., et al., Contribution of diffusion-weighted MR imaging for predicting benignity of complex adnexal masses. Eur Radiol, 2009. 19(6): p. 1544-52.
15. Thomassin-Naggara, I., et al., Dynamic Contrast-Enhanced Magnetic Resonance Imaging: a Useful Tool for Characterizing Ovarian Epithelial Tumors. J Magn Reson Imaging, 2008. 25(1): p. 111-120.
16. Bernardin L, et al. Effectiveness of semi-quantitative multiphase dynamic contrast-enhanced MRI as a predictor of malignancy in comple adnexal masses : radiological and pathological correlation. Eur radiol, 2011. Nov 18.
17. Spencer JA et al. ESUR guidelines for MR imaging of the sonographically indeterminate adnexal mass : an algorithmic approach. Eur radiol, 2010 Jan ;20(1) : p. 25-35
18. Hsieh FY, Bloch DA, Larsen MD et al. A simple method of sample size calculation for linear and logistic regression. Stat Med, 1998. 17: p. 1623-1634.

© 2020 Thomassin-Naggara I et al. *JAMA Network Open.*

Appendix 1 : Lexicon*

Terms	Definitions
Purely cystic mass	Absence of internal enhancement after injection and corresponded a to unilocular cyst or hydrosalpinx, both of which have low T1-weighted and high T2-weighted MR signal intensities
Purely endometriotic mass	Lesion displaying high T1-weighted signal intensity greater or equal to subcutaneous fat, shading on T2-weighted MR sequence and no internal enhancement
Fatty mass without enhanced component	Lesion displaying high T1-weighted signal intensity that disappeared after fat saturation and potentially displaying non-enhancing solid component
Wall enhancement	Enhancement of the wall of a cyst
Bi or multilocularity	A cyst that has two or more septa. A septum is defined as a thin strand of tissue running across the cyst cavity from one internal surface to the controlateral side
Grouped septa	A cyst contains grouped septae if 3 or more septa are close together in a part of the cyst
Thickened regular septa	A smooth septation with a thickness > or equal to 3mm within a cystic tissue.
Solid tissue	As defined by IOTA group, solid tissue displays a positive Doppler flow. Thus, using MR imaging, a solid tissue enhances after gadolinium injection. In adnexal tumors, diffuse wall thickening, normal ovarian stroma and regular septa are not regarded as solid tissue according to IOTA group (13). Thus, solid tissue is either thickened irregular septa, and/or vegetation and/or solid portion (including completely solid mass).
Solid papillary projections	defined by IOTA group as any solid projections into the cyst from the cyst wall with height greater or equal to 3mm
Mixed or purely solid mass	= Solid nodule defined by IOTA group as any solid tissue which is not a wall, a septum or a vegetation. This group comprises completely solid masses
Thickened irregular septa	Focal areas of septal thickening with a thickness >or equal to 3 mm within a cystic tissue.
T2-weighed signal intensity within solid tissue	Signal intensity defined in comparison with adjacent external myometrium
b_{1000}-weighted signal intensity within solid tissue	Signal intensity defined in comparison with serous fluid (i.e., urine in bladder or cerebrospinal fluid [CSF])
Time intensity curve within solid tissue type 1<br>Time intensity curve within solid tissue type 2<br>Time intensity curve within solid tissue type 3	A gradual increase in the signal of the solid tissue, without a well-defined shoulder<br>A moderate initial rise in the signal of solid tissue relative to that of myometrium<br>An initial rise in the signal of solid tissue that was steeper than that of myometrium
Ascites	Fluid in peritoneal cavity
Peritoneal implants	Nodular thickening of the peritoneum that enhances after gadolinium injection
* *From Thomassin-Naggara et al. Radiology 2012 in press*	

© 2020 Thomassin-Naggara I et al. *JAMA Network Open.*

Appendix 2 : ADNEXMR SCORING system (From Thomassin-Naggara et al. Radiology 2012 in press)

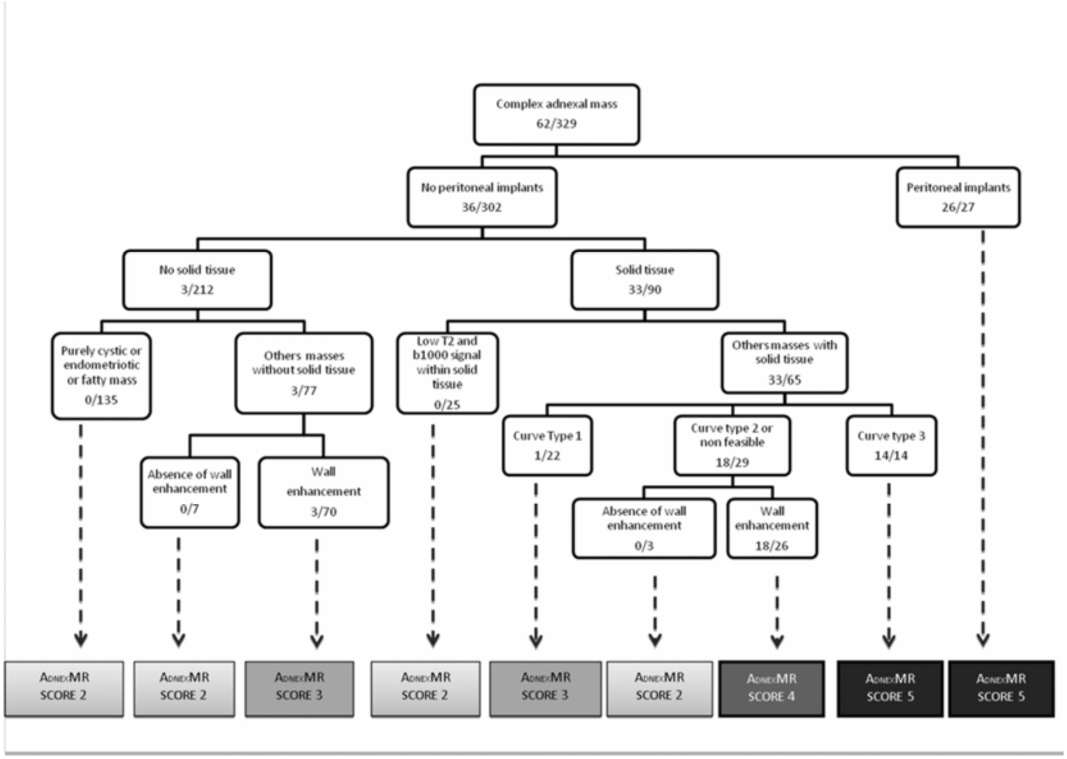

	PLR
ADNEXMR SCORE **1: No mass**	**-**
ADNEXMR SCORE **2 : Benign mass**	**0**
Purely cystic mass<br>Purely endometriotic mass<br>Purely fatty mass<br>Absence of wall enhancement<br>Low b1000 and low T2-weighted signal intensity within solid component	
ADNEXMR SCORE **3: Probably benign mass**	**<0.01**
Curve type 1 within solid tissue<br>Masses without solid tissue (except purely cystic, endometriotic and fatty mass)	
ADNEXMR SCORE **4: Indeterminate MR mass**	**0.1-10**
Curve type 2 within solid tissue	
ADNEXMR SCORE **5 : Probably malignant mass**	**>10**
Peritoneal implants<br>Curve type 3 within solid tissue	

* Only one feature enough to classify in each category

© 2020 Thomassin-Naggara I et al. *JAMA Network Open.*

Appendix 3 : Informed form

Fiche d'information et de consentement dans le cadre de l'étude observationnelle

« Evaluation multicentrique d'une classification diagnostique en IRM pour la caractérisation d'une masse annexielle : EURADMR classification»

Madame, Mademoiselle,

Vous allez réaliser une IRM pelvienne pour caractériser une masse annexielle complexe détectée en échographie. Nous vous proposons de participer à l'étude intitulée : « **Evaluation multicentrique d'une classification diagnostique en IRM pour la caractérisation d'une masse annexielle : EURADMR classification »**

Justification de l'étude :

La caractérisation de la masse annexielle détectée lors de l'échographie que vous avez réalisée va aider votre gynécologue à choisir la meilleure stratégie thérapeutique pour vous. De multiples études de la littérature ont démontré que l'IRM permettait une meilleure caractérisation des masses annexielles par rapport à l'examen clinique et l'échographie.

L'intérêt d'établir une classification diagnostique en IRM des masses annexielles est dans l'avenir de donner au clinicien un appréciation reproductible du risque de malignité. Cette classification n'aura actuellement aucun impact sur votre prise en charge qui sera conforme aux pratiques habituelles recommandées.

Objectifs de l'étude :

L'objectif de cette étude est de valider de façon multicentrique une classification diagnostique en IRM des masses annexielles appelée ADNEX MR SCORING system.

Durée et suivi de l'étude :

Votre participation correspondra à la durée de prise en charge de votre pathologie annexielle. En l'absence de prise en charge chirurgicale, un suivi de deux ans sera réalisé pour vérifier l'absence d'évolutivité de votre masse annexielle.

Risques et contraintes :

Il n'y a aucun risque ou contrainte spécifique à cette étude, l'IRM pelvienne étant réalisée en pratique courante pour les patients dans le service de radiologie de l'Hôpital Tenon. Votre participation n'engendrera aucun frais supplémentaire.

Vos images d'IRM seront anonymisées et envoyées au centre coordinateur de l'étude pour une lecture centralisée

© 2020 Thomassin-Naggara I et al. *JAMA Network Open.*

Effets indésirables et bénéfices attendus :
Les risques de l'étude sont considérés comme faibles à négligeables identiques à ceux engendrés par une IRM pelvienne réalisée en routine clinique

Participation volontaire et droit de se retirer de l'étude :
Votre participation est totalement libre et volontaire. Votre refus sera sans conséquence sur votre prise en charge médicale. Vous pouvez décider à tout moment de vous retirer de l'étude sans donner de raison et sans que votre responsabilité ne soit engagée. Vous continuerez bien sur à bénéficier de tous les soins dont vous avez besoin

Confidentialité et législation :

Les données recueillies demeureront strictement confidentielles. Seuls les responsables de l'étude et éventuellement les autorités de Santé pourront avoir accès à ces données. A l'exception de ces personnes qui traiteront les informations dans le plus strict respect du secret médical, votre anonymat sera préservé. La publication des résultats de l'étude ne comportera aucun résultat individuel.

La base se données de l'étude a été déclarée auprès de la commission Nationale de l'informatique et Libertés (CNIL). Aussi, conformément à la loi « informatique et libertés » (loi 78-17 du 6 janvier 1978 modifiée par la loi 2004-801 du 06 août 2004), vous bénéficiez d'un droit d'accès et de rectification aux informations qui vous concernent.

Conformément à la loi du 9 août 2004, relative à la politique de santé publique et à la protection de personnes en matière de Santé, le projet a été soumis au Comité d'éthique et de Protection des Personnes soumis à une recherche biomédicale ----------- et a reçu un avis favorable.

Vous pouvez également accéder directement ou par l'intermédiaire d'un médecin de votre choix à l'ensemble de vos données médicales en application des dispositions de l'article L.1111-7 du code de santé publique. Ces droits s'exercent auprès du médecin qui vous suit dans le cadre de la recherche et qui connaît votre identité. Vous pouvez à tout moment vous opposer à la transmission de vos données médicales.

Vous pouvez à tout moment demander toute information complémentaire au **Dr.THOMASSIN-NAGGARA** (n° de téléphone : 01 56 01 70 00 (bip 15212))

Après en avoir discuté et avoir obtenu réponse à toutes mes questions, j'accepte librement et volontairement de participer à la recherche décrite ci-dessus. Je suis parfaitement conscient que je peux retirer à tout moment mon consentement à ma participation à cette recherche et cela quelles que soient mes raisons et sans supporter aucune responsabilité.

L'investigateur :
Fait à .... , le
Nom, prénom :
Signature

Le patient :
Fait à .... , le :
Nom, prénom
Signature

© 2020 Thomassin-Naggara I et al. *JAMA Network Open.*

Appendix 6: Informed consent: ENGLAND

Patient information sheet

**Title**	**Multicentre validation of ADNEX-MR classification for characterizing sonographically indeterminate adnexal masses.**
**Short Title**	EurRad
**Study Sponsor**	Imperial College Healthcare NHS Trust
**Version**	1.0
**Date**	September 2012
**PI Name**	<<Insert Local PI Name>>

Dear Madam,
We would like to invite you to take part in a research study. Before you decide whether to take part, it is important for you to understand why the research is being done and what it will involve. Please take time to read the following information carefully. Talk to others about the study if you wish.

Ask us if there is anything that is not clear or if you would like more information. Take time to decide whether or not you wish to take part.

1. **What is the purpose of the study?**

Ultrasound of the pelvis sometimes detects cysts or masses in the pelvis.These are known as adnexal masses. Although most are harmless a few can be more serious including a very small number which may have cancerous changes. If the ultrasound findings are not certain then MRI scanning is often performed to get more information about the mass.
MRI has been found to be very accurate in the diagnosis of indeterminate adnexal masses and provides detailed information about the nature of adnexal masses. MRI is already used routinely to provide more information concerning indeterminate adnexal masses in order to help direct the doctor to the best treatment for the patient. For example, the doctor may need to decide whether surgery will be necessary or not and if surgery is needed, then the type of surgery that will be best for the individual patient.
There have now been many published scientific studies that have reported the ability of MRI to determine the nature of adnexal masses that could not be confidently diagnosed using ultrasound and clinical findings.

A classification system for MRI scan results has been designed which gives a numerical score of 1-5 to indicate the likely risk of the mass being cancer. The importance of the classification is to allow a reliable and reproducible scale which predicts the risk of cancerous change within an adnexal mass. This information will allow the best possible treatment strategy to be chosen for each individual patient. The name of the diagnostic classification is the ‘Adnex-MR score’.

© 2020 Thomassin-Naggara I et al. *JAMA Network Open.*

The purpose of this study is to validate this ADNEX MR score for adnexal masses that were not confidently diagnosed on ultrasound (‘sonographically indeterminate’). The validation of this classification is being undertaken as a multicentre European collaborative trial.
If the scoring system proves to be valid then doctors reporting MRI scans will be able to provide a clear and standardised indication of the level of risk that a mass may be cancerous. This will then, in turn, help determine the most appropriate treatment for patients with this condition.

2. Why have I been chosen?

You have been chosen because your doctor has referred you for an MRI scan of the pelvis, following your recent ultrasound scan, in order to further evaluate the pelvic cyst or mass (an adnexal mass) that was found. We are inviting all patients who have been referred for MRI of an indeterminate adnexal mass to take part in this study.

3. Do I have to take part?

No, it is up to you to decide whether or not to take part. If you choose to take part you will be asked to sign an informed consent form and you will be given a copy to keep, together with this information sheet.
If you do not wish to take part in the study you do not have to give a reason. You will not be disadvantaged in any way, and it will not affect the standard of care you receive. This also applies if you initially decide to take part and then change your mind at a later date.

4.What are the alternatives?

If you decide not to go into this study, you will be offered the MRI scan that your doctor has requested and your MRI scan will be reported in the normal way. You will follow the standard care pathway appropriate for your individual medical needs whether you take part in the study or not .

5.What will happen to me if I take part?

If you decide to take part in the study you will be asked to sign a consent form and your doctor will make sure that you are suitable for entry onto the study. He or she will check that you meet all the requirements for entry and there are no reasons why you should be not take part.
If you are pregnant you will not be able to participate in this study as the scoring system uses information from one scan sequence which uses an injection of a liquid dye called Gadolinium. That sequence is not used in these circumstances as Gadolinium is not recommended in pregnant women. The MRI scan that you will then undergo will be better suited to pregnant patients.

Once these checks have taken place you will then be enrolled onto the study.
Your participation in the study will involve your attending for for a standard MRI scan, as requested by your doctor, which will take approximately 45 minutes.
Information about the standard MRI scan will be given to you by the imaging department when your appointment is made. Your MRI scan will be reported and the results will be made available to your doctors. Following discussion with your doctor,

© 2020 Thomassin-Naggara I et al. *JAMA Network Open.*

you will proceed to treatment according the standard of care for your condition. This is your normal clinical care.
If you consent to participate in the study then your scans will be reviewed and scored by the radiologists participating in the research. In addition to the routine clinical report they will use a predefined scoring system to categorise your scan appearances. The score uses a 5 point scale to indicate the likelihood that the cysts or mass in your pelvis might be cancerous. Similar scoring systems have been used for some time in breast imaging. Following the scan we would collect information to see how effective the scoring system was at predicting the correct diagnosis.
If you have surgery we will collect information from the surgeon and the pathologists looking at sample in order to know the final diagnosis. If you do not have surgery we will collect information from any follow up appointments, scans or surgery that you have over the next two years. This information will be collected by the researcher at your hospital who will review your clinical notes and imaging studies that you may undergo during that period of time.
By consenting to the study, you give us permission to use the proposed scoring system to evaluate your scan and then to collect information about what happens to you after the scan for a period of two years. The research team will compare the Adnex-MR score with the outcome of your surgery or any follow-up appointments

6. What do I have to do?

Should you become a participant in the study, then you will be required to attend your scheduled visit for MRI. If you decide to take part in this study, you should not be involved in any other imaging trial at the same time. No other study procedure is required. However, we will follow your progress to record whether you have surgery or not and record the results of any surgery or follow-up for a two year period.

7. Will my taking part in this study be kept confidential?

Yes. All information which is collected about you during the course of the research will be kept strictly confidential. If you consent to take part in the research, the people conducting the study will abide by the Data Protection Act 1988, and the rights you have under this Act.
If you join the study, some parts of your medical records and the data collected for the study will be looked at by authorised personnel from your treating centre and authorised personnel from <<Name of Hospital taking part in the study>>. It may also be looked at by representatives of regulatory authorities and other authorised personnel from your trust, to check that the study is being carried out correctly. All will have a duty of confidentiality to you as a research participant and nothing that could reveal your identity will be disclosed outside the research site. The information that we will collect for the purposes of this study will be entered on a paper form on which your name will be anonymised, although at each centre, the research team will securely store information that will be able to link you to the anonymisation number. The anonymised data will be entered on an electronic database. MRI scans will be reported on the clinical system normally. An anonymised copy of the scan will be stored on CD for two radiologists to score your scan, in order to test the reproducibility of the score between different radiologists. These radiologists may be

© 2020 Thomassin-Naggara I et al. *JAMA Network Open.*

other investigators in the UK and in Europe as part of the multicentre collaboration. All data will be stored in a locked and dedicated room at <<Imperial College Healthcare NHS Trust>> which will only be accessible by authorised personnel.

8. Expenses and payments

If you choose to participate in this research study, you will not have any additional visits to the hospital other than those required for your routine care. Unfortunately, we are unable to reimburse any travel costs that may be incurred for attending your MRI scan. If this is a problem for you, then please discuss this with your study doctor.

9. What are the other possible disadvantages and risks of taking part?

There is no specific risk related to taking part in this study, as the study focuses on testing a diagnostic classification of the standard MRI scan that your doctor has requested. You will not undergo any different or additional scan or test for the purposes of the study. The hospital department which will undertake the MRI scan performs this type of scan routinely.

10. Risks to an Unborn Child

If at the time of any imaging procedures you suspect you may be pregnant, then you must inform your doctor, who will advise and help you. An MRI scan may be done in pregnancy but the liquid dye that is used as standard (gadolinium) would not be used and you would not be eligible to take part in the study.

11. What happens if there is a problem?

We would not expect you to suffer any harm or injury because of your participation in this study, as the study does not involve any change from your normal care. Regardless of this, if you wish to complain or have any concerns about any aspect of the way you have been approached or treated during the course of this study, the normal National Health Service complaints mechanisms should be available to you.

Please contact Patient Advisory Liaison Service (PALS) if you have any concerns regarding the care you have received, or as an initial point of contact if you have a complaint. Please telephone <<Insert PALS Telephone Number>> or email <<Insert PALS Email>> you can also visit PALS by asking at any hospital reception.

12. What if relevant new information becomes available?

Sometimes during the course of a research project new information becomes available about the diagnostic test being studied. This is very unlikely to happen in this type of study. However, if this happens, your consultant will tell you about it and discuss whether you want to take part in the study. If you decide to withdraw, your study doctor will make arrangements for your care to continue.

13. What will happen if I don’t want to carry on with the study?

You are free to withdraw from the study at any time and do not have to give a reason. Your future treatment will not be affected and your doctor will discuss this with you. We would like your permission to continue to receive information on your progress. If

you decide that we may have no further information from you for the study, we will need to use the data collected up to the time of your withdrawal.

14. What are the possible benefits of taking part?

You may not benefit directly by taking part in this trial. However, it is hoped that the information from this study will allow doctors in the future to give better information to patients with this kind of problem allowing them to make informed choices regarding their care,

15. What happens when the research study stops?

Following completion of your MRI scan, you will have ended your active study participation. We will then monitor your treatment and follow-up either until you have surgery for the adnexal mass or, if you do not have surgery, we will monitor any further imaging tests that you have of the adnexal mass, such as future ultrasound or MRI scans. The reason for this follow-up monitoring is to see whether the scoring classification was correct or whether it was wrong.
When the results of the study are available a summary can be provided to you upon request.

16. Who is organising and funding the research and where was it reviewed?

This is an investigator-initiated study. {Dr Isabelle Thomassin-Naggara of Hopital is the Chief Investigator}. Imperial College Healthcare NHS Trust is sponsoring the study. The doctors and other members of the clinical research teams are not being paid for participating in this study.

17. Who has reviewed the research?

This study has been through a peer review process. A peer review involves the examination of an author's work by other experts in the same field. These referees each return an evaluation of the work which may include suggestions of improvements if necessary.
Your local NHS Trust has been given approval for the study to take place at your hospital. The study has also been reviewed by a Research Ethics Committee.

18. Who can I contact for further information?

For questions about the study you may contact your research doctor or nurse, the contact details are below:

Dr <<Insert Doctor Name>>
Telephone: <<Insert Doctor Telephone Number>>

Or;

Research Nurse <<Insert Research Nurse Name>>

© 2020 Thomassin-Naggara I et al. *JAMA Network Open.*

Telephone: <<Insert Research Nurse Telephone

Number>> For information about your disease:

??Ovarian cancer helpline?

CancerHelp UK provides general information for patients about cancer and its treatment on their website, www.cancerhelp.org.uk. The information can also be available over the phone by contacting a specialist research nurse on the 020 7061 8355 or free-phone 0808 800 4040.

We are Macmillan provides support and counselling to help people living with cancer, information can be found on their website, www.macmillan.org.uk, or by free- phone on 0808 808 2020

© 2020 Thomassin-Naggara I et al. *JAMA Network Open.*

eAppendix 3. Population Selection Criteria

**Inclusion Criteria**<br>- Patient ≥ 18 years old<br>- Sonographically indeterminate adnexal mass<br>- Informed consent<br>**Exclusion Criteria**<br>- Pregnancy (relative contraindication for gadolinium injection)<br>- Pacemaker, ferromagnetic materials, or foreign body at risk of mobilization or any other contraindication to MR imaging.<br>- Intolerance to gadolinium contrast agents, or severe renal insufficiency (GFR <30 ml/min/1.73m^2).
**Ultrasound findings (Quoted 7 points in all criteria)** (950/1194 (79.6%) patients had a score ≥5/7)<br>1- Side<br>2- Size<br>3- Origin<br>4- Presence of solid component<br>5- Ipsilateral ovarian parenchyma<br>6- Positive Doppler<br>7- Pelvic Fluid

© 2020 Thomassin-Naggara I et al. *JAMA Network Open.*

eAppendix 4. Methodological Details

2A : Technical MR acquisition
Each patient underwent a routine pelvic MR imaging (1.5T or 3T), including morphological sequences (T2, T1 with and without fat suppression and T1 after gadolinium injection) and functional sequences (perfusion and diffusion-weighted sequences). If at the time of inclusion and MRI scan, the adnexal mass has disappeared on T2 and T1 weighted sequences, functional sequences and gadolinium injection were not mandatory. Each centre used functional sequences with the following criteria:
DCE MR sequence: 3D isotropic**,** Delay between the beginning of the sequence and injection: 1 minute**,** Total duration after injection: 3min, Slice 3mm no gap (1 slice on 2 with the exact same location than T2)**,** Spatial resolution: 3mm**,** Temporal resolution < 15s**,** Box size: 15cm
DWI sequence **:** Exactly same slice thickness as the T2**,** b1000-b1200 (to ensure that urine in bladder is black)
Technical requirements for DCE-MR sequence were checked at a preliminary period by an MR scientist.
2B Quality Assurance: The first patient from each centre was sent to main investigator to check if the acquisition protocol was correct especially regarding technical requirements for DCE MR sequence and DW sequence (supplementary material 1). Subsequent quality assurance was then undertaken by the site PI to ensure on-going adherence to the study imaging protocol.
2C Reader training: During study set-up, a session of 30 anonymized DICOM cases (previously acquired before the beginning of the study) were downloaded for a training session to learn how to apply the MR score for all teams participating in the multicentre validation. Readers were trained in using the standardized lexicon (suppl. table 2) for interpretation.

eTable 1. Lexicon and Supplementary Material with Detailed Analysis of Adnexal Lesions Based on Morphological Characteristics

**Terms and Definitions**	**Per lesion study results (n=1502)**
**Adnexal origin**	Adnexal origin corresponds to all lesion developed on ovary, tube or mesosalpinx. Adnexal origin was described in 91.3% (1372/1502). Twelve adnexal masses were misclassified as non-adnexal (8 benign masses classified as score 1 non suspicious and 4 malignant tuboovarian masses rated as score 1 suspicious). Ten (83.3%) of these 12 misclassified adnexal masses were larger than 5cm. Twenty eight non-adnexal masses misclassified as adnexal masses. Eighteen non adnexal masses were rated score 2 or 3 and all corresponded to benign masses. Nine non adnexal masses were rated as adnexal masses scored 4 or 5 and corresponded to 5 extra adnexal cancers, 3 uterine leiomyoma and one schwanoma.
**Purely cystic mass**<br>Absence of internal enhancement after injection and corresponding to a unilocular cyst or hydrosalpinx, both of which have low T1-weighted and high T2-weighted MR signal intensity of internal cyst content	Purely cystic mass was described in 27.4% (411/1502) and were all benign apart from one serous carcinoma (however, due to peritoneal carcinomatosis, this patient was assigned MR score 5). This group corresponds mainly to serous cystadenoma (n=148), functional cyst (n=134), pelvic inflammatory disease or hydrosalpinx (n=48), peritoneal cyst (n=27), paraovarian or paratubal cyst (n=16), others (n=38).
**Purely endometriotic mass**<br>Lesion displaying high T1-weighted signal intensity ≥ to subcutaneous fat, shading on T2-weighted MR sequence and no internal enhancement	Purely endometriotic mass was described in 12.4% (187/1502) and were all benign apart from one borderline serous tumour. This description was mainly used for endometrioma (n=154) or endometriotic hematosalpinx (n=6). Other diagnoses were functional cyst (n=15), pelvic inflammatory disease or hydrosalpinx (n=7), others (n=5)
**Fatty mass without enhanced component**<br>Lesion displaying high T1-weighted signal intensity that disappeared after fat saturation and potentially displaying non-enhancing solid component	Purely fatty mass was described in 6.4% (97/1502) and all corresponded to benign lesions with mature cystic teratoma in 95.9% (93/97). The four other lesions were serous cystadenoma, functional cyst and indeterminate due to adnexal torsion.
**Wall enhancement**<br>Enhancement of the wall of a cyst	No wall enhancement was reported in 22.6% (339/1502) and were all benign lesions except one serous borderline tumour (the same false negative described in purely endometriotic group). This feature was described mainly in endometrioma (n=112), mature cystic teratoma (n=55), functional cyst (n=53), serous benign cystadenoma (n=50) and peritoneal cyst (n=22).

© 2020 Thomassin-Naggara I et al. *JAMA Network Open.*

**Bi- or multilocularity**<br>A cyst that has two or more septa. A septum is defined as a thin strand of tissue running across the cyst cavity from one internal surface to the contra-lateral side<br>**Grouped septa**<br>A cyst contains grouped septae if three or more septae are close together in one part of the cyst<br>**Thickened regular septa**<br>A smooth septation with a thickness ≥ 3mm within a cystic tissue.	Bi- or multiloculate cyst **without solid tissue** was described in 12.7% (191/1502) of adnexal masses, including only five malignant tumours (one tubal cancer, two borderline mucinous tumours, one invasive mucinous carcinoma, and one peritoneal carcinoma). Thickened smooth septa were present in three malignant epithelial tumours but also described in 57 benign lesions (PPV = 5%). In bi- or multiloculate cysts with thin smooth septa and without solid tissue (n=131), the PPV for malignancy was 1.5% (2/131).
**Solid tissue**<br>A solid tissue enhances after gadolinium injection. In adnexal tumours, diffuse wall thickening, normal ovarian stroma and regular septa are not regarded as solid tissue according to IOTA group (13). Thus, solid tissue is either thickened irregular septa, and/or vegetation and/or solid portion (including completely solid mass).<br>**Solid papillary projections** are defined as any solid projections into the cyst from the cyst wall with height ≥ 3mm<br>**Mixed or purely solid mass** is defined as any solid tissue which is not a wall, a septum or a vegetation. This group comprises completely solid masses<br>**Thickened irregular septa**<br>Focal areas of septal thickening with a thickness ≥ 3 mm within a cystic tissue.	No Solid tissue (n=911, 60.6%) was reported in 902 benign lesions and 9 malignant tumours. Solid tissue was present in 591 tumours including 323 benign and 268 malignant tumours. Solid papillary projections were described in 170 tumours (86% were epithelial tumours at final diagnosis (146/170)) with a PPV of malignancy of 55.8% (95/170) (including 35 borderline and 60 invasive). Mixed masses were described in 257 tumours with a PPV of malignancy of 62.6% (161/257). Purely solid masses were described in 211 tumours with a PPV of malignancy of 32.3% (66/211). Thickened irregular septa were described in 74 masses with a PPV of malignancy of 66.2% (49/74). Benign tumours with thickened irregular septa (n=25) were mainly epithelial tumours (n=10), mature cystic teratoma (n=5), pelvic inflammatory disease or hydrosalpinx (n=5)
**T2-weighted signal intensity within solid tissue**<br>Signal intensity defined in comparison with adjacent external myometrium<br><br>**b_{1000}-weighted signal intensity within solid tissue**<br>Signal intensity defined in comparison with serous fluid (i.e., urine in bladder or cerebrospinal fluid [CSF])	Solid tissue with low T2 and low DW signal intensity was present in only two malignant tumours (one borderline serous tumour and one metastasis) (false negative rate 1.9% (2/105)). However, when solid tissue was low only on T2W sequence, there were 19 malignant tumours (false negative rate 20.8% (19/91)) and when solid tissue was low only on DW sequence, there were 10 malignant tumours (false negative rate 18.8% (10/53)) .
**Time intensity curve within solid tissue**<br>**Type 1:** A gradual increase in the signal of the solid tissue, without a well-defined shoulder<br>**Type 2:** A moderate initial rise in the signal of solid tissue relative to that of myometrium<br>**Type 3:** An initial rise in the signal of solid tissue that was steeper than that of myometrium	When solid tissue enhanced according a time intensity curve type 3, malignant tumours were encountered in 85.6% (143/167). False positives were 10 uterine leiomyomas, five mature cystic teratomas, two Brenner tumours, two cystadenofibromas, one functional cyst, one schwannoma, one struma ovarii, one benign cystadenoma and one ovarian fibroma. Invasive malignant tumours enhanced according a time intensity curve type 3 in 59.4% (138/232), type 2 in 32.3% (75/232), type 1 in 2.1% (5/232). In the fourteen remaining invasive adnexal tumours, time intensity curve was not feasible in eight cases and no solid tissue was detected in six.

© 2020 Thomassin-Naggara I et al. *JAMA Network Open.*

Terms and Definitions	Per patient study results (n=1130)
**Peritoneal implants**<br>Nodular thickening of the peritoneum that enhances after gadolinium injection	Peritoneal implants were described in 72/1130 patients with four false positive cases (two pelvic inflammatory disease, one functional cyst and one leiomyoma) (LR+ = 77.63 95% CI =28.65-210.37).
**Pelvic fluid**<br>Presence of fluid in peritoneal cavity	Pelvic fluid was described in 433/1130 patients (38.3%) including 287 with benign and 146 with malignant lesions (LR+ = 2.04 95% CI $_{=2.04-2.64}$). When there was abdominopelvic fluid (4.6%, n=52/1130), all lesions were malignant apart from four (two serous cystadenomas, one pelvic tuberculosis and one ovarian fibroma) (LR+= 54.8, 95% CI =19.99-150.25).

© 2020 Thomassin-Naggara I et al. *JAMA Network Open.*

eTable 2. Diagnostic Values of Different MRI Features

	LR+	LR-	Prevalence (n=1502)	Rate of malignancy (PPV)	False negatives	Kappa (experienced/junior)
Purely cystic mass (n=411)	0.01 (0-0.08)	1.50 (1.44-1.56)	27.4%	0.2% (1)	1 serous carcinoma*	0.73 (0.68-0.77)
Purely endometriotic mass (n=187)	0.02 (0-0.17)	1.17 (1.15-0.20)	12.4%	0.5% (1)	1 borderline serous tumour^	0.82 (0.78-0.87)
Purely fatty mass (n=97)	0	1.08 (1.07-1.10)	0	0%	-	0.72 (0.65-0.79)
Absence of wall enhancement (n=339)	0.01 (0-0.09)	1.38 (1.33-1.43)	22.6%	0.3% (1)	1 borderline serous tumour^	0.43 (0.39-0.47)
Bi- or multiloculate cyst without solid tissue (n=191)	0.12 (0.05-0.29)	1.16 (1.13-1.19)	12.7%	2.6% (5)	2 borderline mucinous tumour**<br>1 invasive mucinous carcinoma**<br>1 tubal cancer<br>1 peritoneal carcinoma	0.63 (0.59-0.67)
- Thick septa (n=60)	0.23 (0.07-0.74)	1.04 (1.02-1.06)	4%	5% (3)		
- Thin septa (n=131)	0.07 (0.02-0.28)	1.11 (1.09-1.13)	8.7%	1.5% (2)		
Absence of solid tissue (n=911)	0.04 (0.02-0.08)	3.67 (3.33-4.04)	60.6%	1% (9)	3 borderline tumours<br>3 ovarian carcinoma<br>2 tubal carcinoma<br>1 peritoneal carcinoma	0.81 (0.78-0.84)
Solid tissue with lowT2 and low DW signal (n=105)	0.09 (0.02-0.35)	1.08 (1.06-1.11)	7%	1.9% (2)	1 borderline serous tumour<br>1 ovarian metastasis	0.70 (0.664-0.735)
Solid tissue with TIC type 1 (n=180)	0.32 (0.18-0.56)	1.11 (1.07-1.15)	11.9%	6.7% (12)	7 borderline cystadenoma<br>1 ovarian cystadenocarcinoma<br>1 Granulosa<br>3 ovarian metastasis	0.66 (0.617-0.71)
Solid tissue with TIC type 2 (n=223)	3.9 (3.08-4.86)	0.69 (0.63-0.76)	14.8%	46.6% (104)		
Solid tissue with TIC type 3 (n=167)	26.3 (17.4-39.7)	0.49 (0.44-0.56)	11.1%	85.6% (143)		

* because of peritoneal carcinomatosis, this patient was assigned O-RADS Score 5
**Displays thickened regular septa (3 malignant tumours of the 60 lesions with thickened smooth septa)
(95%CI)
^ these were the same lesions

© 2020 Thomassin-Naggara I et al. *JAMA Network Open.*

eFigure. Score Performance Among Senior and Junior Readers

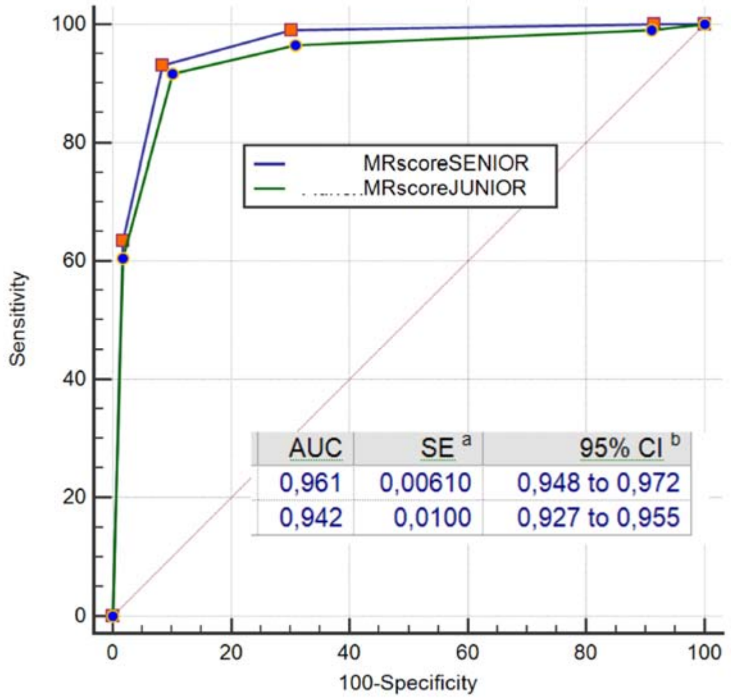

© 2020 Thomassin-Naggara I et al. *JAMA Network Open.*
